# Prognostic value of the Duke Activity Status Index for preoperative cardiac risk stratification: an international pooled cohort study

**DOI:** 10.1016/j.eclinm.2026.104015

**Published:** 2026-06-11

**Authors:** Duminda N. Wijeysundera, Brian H. Cuthbertson, Emmanuelle Duceppe, Tom E.F. Abbott, Rupert M. Pearse, Paul S. Myles, Keying Xu, Julian F. Daza, Calvin Diep, Leandra A. Amado, Angela Jerath, Bernhard Riedel, Daniel I. McIsaac, FIT After Surgery Investigators and METS Investigators, S. Abdellatif, M. Abolfathi, G.L. Ackland, T. Ahmad, E. Al Azazi, M. Ali, S.M.H. Alibhai, S. Allen, A. Ambosta, M. Alsaif, N. Ami, L. Andrews, S. Au, S. Avramescu, G. Back, H. Bagry, T. Barnes, S. Bates, W.S. Beattie, N. Beauchamp, C. Beilstein, R. Belliard, E. Black, A. Bodger, P. Bodger, M.F. Boko, C. Bolger, F. Bonazza, B. Borg, M. Bosch, D. Bramley, J. Brannan, R.H. Breau, A. Brown, R. Brull, K. Brunello, D. Campbell, A.M. Carrera, M. Celinski, V. Chan, S. Charummoottil, I. Chergui, T.R. Chesney, S. Choi, H.A. Clarke, A. Collingwood, H. Collins, C. Corriea, T. Creary, C. Crescini, B.L. Croal, J. Dale-Gandar, P. Dalley, M. Davis, L. Day, A. Dent, D. Dillane, J. Dimech, J. Dion, K. Dobson, J. Douglas, S. Drozdz, L.M. Drudi, D. Dumerton, M. Edwards, N.Y. Edwards, S. Ehtesham, H. El Beheiry, A. Elliott, M. Ellis, Y. Essaji, K. Everingham, C. Farrington, A. Ferenc, H. Filipe, A. Fleet, K. Flores, S. Gabriel, S. Gagne, L. Gallego-Paredes, S. Gandotra, M. Gertsman, G. Godsall, A. Goel, K. Golder, J. Grant, J.T. Granton, K. Greaves, J. Green, M.P.W. Grocott, U. Gurunathan, A Gutierrez Del Arroyo, K. Hagen, A. Hall, C. Hanley, R. Haslop, K. Higgie, G. Hillis, E. Hladkowicz, H. Houston, Y. Hu, A. Hunt, S. Hurford, J. Hutton, H. Ismail, S. Jack, E. Jacobsohn, S. Jain, S. James, R. Jang, M. Januszewska, A. Jason-Smith, S. Jhanji, M. Karizhenskaia, K. Karkouti, L. Kaustov, K. Kenchington, A. Kennedy, E. Kennedy, C. Keown-Stoneman, T. Kerelska, R.K. Kerridge, S. Khandadashpoor, C.J. Kim, C. King, Y. Kirabiyik, M. Koutra, K. Kuang, J. Kumar, J. Kunasingam, S. Kynaston, K.S. Ladha, M. Lalu, H. Lawrence, G. Lebovic, L. Lee, S. Lee, K. Leslie, D. Levett, R. Lifford, H. Lindsay, G. Lorello, M. Lorimer, L. Loughney, M. Louridas, M. Lum, V. Lyon, A. Maccormick, D. Macdonald, N. Macdonald, S.Y. Macdonell, S. Macklin, J. Malherbe, A. Malik, M. Mamdani, F. Marshall, D. Martin, P. Masel, G. Mattina, C.D. Mazer, D. Mcallister, C.J. McCartney, S.A. McCluskey, N. McMillan, J. Mcneil, M. Melo, A. Melville, R. Miller, J.F. Morales, A. Murmane, L. Navarra, E. Niebrzegowska, S. Nnorom, P. Oh, S. Olliff, T.W. Painter, M.L. Pakats, M. Parotto, J. Pazmino-Canizares, S.M. Pereira, M. Phull, M. Pinto, K. Pirie, S. Pitt, H. Poonawala, J. Pretto, M. Puts, A. Raj, N. Rajapakse, R. Raobaikady, A. Reyes, C. Richard, M. Rooms, L. Ruo, S.L. Russell, F. Saad, K. Salmon, J. Samuel, R. Sara, L. Seaward, P.E. Serrano, S. Shaheen, T. Short, M.A. Shulman, N. Siddiqui, H. Sivakumar, P. Sivalingam, B. Smethurst, E. Smith, P. Somascanthan, A.M. Southcott, R. Spence, M. Stanbrook, K. Steele, R.C.M. Stephens, C. Stonell, D. Sussman, N.F. Syeda, J. Tai, N. Tantony, H. Taylor, N.C. Terblanche, M. Tessier, B. Thompson, H. Thompson, M. Thorleifson, K.E. Thorpe, A. Tippett, R. Tod, E. Torres, M. Towns, O. Tronstad, B. Tyrell, J. Van Der Westhuizen, J. Van Vlymen, F. Vandenbroucke-Menu, E. Vowotor, S. Wallace, M. Wasowicz, E. Waymouth, A.C. Wei, J. Whalley, H.C. Wijeysundera, C. Wilde, C. Wong, E. Wright, J. Wu, K. Xu, A. Yagnik, S. Yagnik, M. Yang, A. Zahavich, K. Zarnke

**Affiliations:** a Department of Anesthesia, St. Michael’s Hospital – Unity Health Toronto, Toronto, Ontario, Canada; b Department of Anesthesiology and Pain Medicine, Temerty Faculty of Medicine, University of Toronto, Toronto, Ontario, Canada; c Strategic Partnerships in Health Excellence, Research, and Engagement, Unity Health Toronto, Toronto, Ontario, Canada; d Department of Critical Care Medicine, Sunnybrook Health Sciences Centre, Toronto, Ontario, Canada; e Departments of Medicine, Centre Hospitalier de l’Universite de Montreal and University of Montreal, Montreal, Quebec, Canada; f Faculty of Medicine and Dentistry, Queen Mary University of London, London, UK; g Department of Anaesthesiology and Perioperative Medicine, Alfred Hospital, Melbourne, Victoria, Australia; h Department of Anaesthesiology and Perioperative Medicine, School of Translational Medicine, Monash University Melbourne, Victoria, Australia; i Division of General Surgery, Department of Surgery, Temerty Faculty of Medicine, University of Toronto, Toronto, Ontario, Canada; j Departments of Anesthesiology and Pain Medicine, University of Ottawa and The Ottawa Hospital, Ottawa, ON, Canada; k Department of Anesthesiology and Pain Medicine and Sunnybrook Research Institute, Sunnybrook Health Sciences Centre, Toronto, Ontario, Canada; l Department of Anaesthesia, Perioperative Medicine, and Pain Medicine, Peter MacCallum Cancer Centre, Melbourne, Victoria, Australia; m Department of Critical Care and the Sir Peter MacCallum Department of Oncology, University of Melbourne, Victoria, Australia

**Keywords:** Functional capacity, Duke Activity Status Index, Cardiac risk stratification, Non-cardiac surgery, Prognosis, Postoperative complications

## Abstract

**Background:**

Guidelines recommend structured self-reported functional capacity assessment for preoperative cardiac risk stratification, including the Duke Activity Status Index (DASI). However, evidence supporting its incremental prognostic value beyond established risk factors remains limited. We evaluated the prognostic performance of the DASI using pooled data from two prospective cohorts.

**Methods:**

We conducted a pooled cohort analysis of adults undergoing elective major non-cardiac surgery enrolled in the Measurement of Exercise Tolerance before Surgery (METS) and Functional Improvement Trajectories After Surgery (FIT After Surgery) studies, including data collected between March 2013 and April 2023. Before surgery, participants completed the Duke Activity Status Index (DASI), a structured 12-item questionnaire based on daily physical activities, and underwent routine preoperative biomarker measurement. The primary outcome was 30-day major cardiac complications (myocardial infarction or non-fatal cardiac arrest) or death. The secondary outcome was all-cause major complications. Hierarchical logistic regression assessed the incremental prognostic value of the DASI beyond age, Revised Cardiac Risk Index (RCRI), and natriuretic peptide concentration. Prognostic performance was evaluated using the likelihood ratio test (LRT), fraction of new predictive information, net reclassification improvement, c-index, calibration plots, and decision curve analysis.

**Findings:**

Among 3485 patients, 3.6% (n = 126) experienced the primary outcome and 19% (n = 647) experienced the secondary outcome. The DASI provided prognostic information beyond age, RCRI, and natriuretic peptide concentration for the primary outcome (LRT p = 0.009), and beyond age, sex, and surgery type for the secondary outcome (LRT p < 0.001). Inclusion of the DASI improved prognostic performance across multiple metrics, but overall discrimination of the final models remained modest (c-index 0.70–0.71), with limited net clinical benefit. Predicted risk associated with a given DASI score varied substantially by age, RCRI, and natriuretic peptide concentration, supporting interpretation of the DASI as a continuous prognostic marker rather than a dichotomous screening test.

**Interpretation:**

The DASI provides incremental prognostic information for preoperative cardiac risk assessment beyond guideline-recommended predictors. Its prognostic implications are modest, context-dependent, and best interpreted as a continuous prognostic marker alongside established risk factors, rather than as a stand-alone threshold-based tool.

**Funding:**

Canadian Institutes of Health Research; PSI Foundation; and the Elizabeth A. and Richard J. Currie, O.C. Chair in Translational Anesthesia Research at St. Michael’s Hospital and the University of Toronto; The Ottawa Hospital Academic Medical Organization Innovation Fund; Heart and Stroke Foundation of Canada; Ontario Ministry of Health and Long-Term Care; Ontario Ministry of Research, Innovation and Science; UK National Institute of Academic Anaesthesia; UK Clinical Research Collaboration; Australian and New Zealand College of Anaesthetists; Monash University.

**Research in context**

**Evidence before this study**

To assess prior evidence on the prognostic value of the Duke Activity Status Index (DASI) for preoperative risk stratification, we searched PubMed using the terms [(“*DASI*” OR “*Duke Activity Status Index*”) AND “*surgery*”] for studies published before 10 December 2025, and reviewed reference lists of relevant reviews and international practice guidelines. Existing primary evidence for the DASI has largely come from the Measurement of Exercise Tolerance before Surgery (METS) study, which demonstrated an association between DASI scores ≤34 and increased risk of postoperative myocardial infarction and death. However, this evidence was based on a limited number of outcome events and did not establish whether the DASI provides meaningful incremental prognostic information beyond guideline-recommended clinical indices and cardiac biomarkers. A 2025 systematic review similarly concluded that the DASI is associated with perioperative outcomes, but more robust multicentre evidence is needed to define its incremental prognostic value and clinically actionable risk thresholds.

**Added value of this study**

To our knowledge, this pooled cohort analysis provides the most comprehensive evaluation to date of the prognostic performance of the 12-item DASI in major non-cardiac surgery. The DASI provided incremental prognostic information for 30-day major cardiac complications or death when added to models that included age, the Revised Cardiac Risk Index, and preoperative natriuretic peptide concentration, as demonstrated across multiple complementary performance metrics. Importantly, the DASI was best interpreted as a continuous risk marker rather than a dichotomous screening test based on a single threshold (e.g., ≤34). The absolute cardiac risk associated with a given DASI score varied substantially according to age, comorbidity burden, and natriuretic peptide status, allowing clinically interpretable and context-specific interpretation of DASI values when anchored to actionable 30-day cardiac risk levels.

**Implications of all the available evidence**

Structured self-reported functional capacity assessment using the 12-item DASI provides prognostic information not captured by traditional clinical risk indices or cardiac biomarkers alone. Its prognostic implications are continuous and context-dependent rather than adequately summarised by a single universal threshold such as a DASI score ≤34. Interpretation of the DASI should therefore account for the broader clinical context, including age, comorbidity burden, and natriuretic peptide status. These findings support integrating the DASI alongside established predictors to enable more precise perioperative cardiac risk estimation, inform postoperative monitoring strategies, guide selection for additional preoperative testing or specialist consultation, and strengthen shared decision-making.

## Introduction

Assessment of functional capacity is a cornerstone of preoperative cardiac risk evaluation for patients undergoing major non-cardiac surgery.^1^^,^^2^ In routine practice, this is usually based on a clinician’s subjective judgment during an unstructured preoperative interview. However, such subjective assessments correlate poorly with objectively measured cardiopulmonary fitness,^3^ and provide little incremental prognostic value for postoperative cardiac complications.^3^, ^4^, ^5^

In recognition of these limitations, international guidelines increasingly recommend structured questionnaires to estimate functional capacity.^1^^,^^2^ A recent scoping review highlighted substantial heterogeneity in how such instruments have been evaluated in the perioperative setting, with limited evidence on their incremental prognostic value beyond established clinical factors.^6^ Among the available tools, the Duke Activity Status Index (DASI) and the MET-REPAIR questionnaire have been most widely studied, each showing associations with postoperative cardiac complications in large international cohorts.^3^^,^^7^ The DASI offers several advantages, including simplicity, feasibility, and availability in several validated translations,^8^, ^9^, ^10^ supporting its application across diverse global healthcare settings. The 2024 American College of Cardiology (ACC) and American Heart Association (AHA) guidelines recommend a DASI threshold of ≤34 to identify patients who warrant further risk assessment before non-cardiac surgery.^1^ However, this threshold was derived from a limited number of outcome events (death or myocardial infarction) in the Measurement of Exercise Tolerance before Surgery (METS) study, raising uncertainty about its robustness and generalisability.^11^ More rigorous evidence is therefore needed to clarify how the DASI should be interpreted within contemporary guideline-based perioperative cardiac risk assessment frameworks, including whether it meaningfully improves prognostic risk estimation beyond established predictors.

The prognostic value of the DASI has now been evaluated in two large multicentre cohorts: the METS study and the Functional Improvement Trajectories After Surgery (FIT After Surgery) study.^3^^,^^12^ Both studies enrolled adults undergoing elective inpatient non-cardiac surgery, administered the DASI preoperatively, and implemented standardised postoperative troponin surveillance, blinded outcome adjudication, and 30-day outcome assessment. Pooling these cohorts provides a unique opportunity to generate more precise estimates of the prognostic value of the DASI, determine whether it improves prognostic risk estimation beyond guideline-recommended factors, and examine how DASI scores relate to clinically relevant absolute risk thresholds across different clinical contexts.

## Methods

### Study design

We conducted a pooled cohort analysis of de-identified individual-level data from the METS and FIT After Surgery studies.^3^^,^^12^ Both studies obtained ethics approval at participating centres, all participants in both studies provided informed consent, and the pooled analysis was approved by the Unity Health Toronto Research Ethics Board (REB file 24–236). The analysis included METS participants who underwent surgery and FIT After Surgery participants who underwent surgery and consented to secondary data use.

METS enrolled patients at 24 centres in Canada, Australia, New Zealand, and the United Kingdom between March 2013 and March 2016. Eligible participants were aged ≥ 40 years, scheduled for elective non-cardiac surgery under general or regional anaesthesia with an anticipated hospital stay of at least one night, and had one or more risk factors for coronary artery disease or cardiac complications. FIT After Surgery enrolled patients at 17 centres in Canada between December 2019 and April 2023. Eligible participants were aged ≥ 65 years and scheduled for elective non-cardiac surgery with an anticipated hospital stay of at least two nights. Additional details for both cohorts are provided in the Appendix (Supplementary Methods).

Before surgery, participants in both cohorts completed the DASI and underwent testing for N-terminal pro-B-type natriuretic peptide (NT-proBNP) or B-type natriuretic peptide (BNP). Troponin was measured daily until postoperative day three or hospital discharge, whichever occurred first. Patients with elevated troponin also underwent 12-lead electrocardiography. Myocardial injury and infarction were determined by an independent adjudication committee, blinded to DASI and natriuretic peptide results.^13^^,^^14^ Other complications (definitions provided in Supplementary Table S2 in the Appendix) were assessed daily, and the complication with the highest level of severity was further classified as mild, moderate, severe, or fatal using a modified International Surgical Outcomes Study (ISOS) classification.^15^ Major complications were defined as those of moderate or greater severity.^3^ After discharge, vital status and complications were assessed at 30 days postoperatively.

### Measures

The DASI (Supplementary Table S1, Appendix) is a 12-item structured questionnaire that quantifies functional capacity based on self-reported daily physical activities. Scores range from 0 to 58.2, with higher values indicating greater functional capacity. The DASI is a valid measure of cardiopulmonary fitness in surgical patients.^3^ To minimize bias, clinicians involved in perioperative care were blinded to DASI scores.

### Outcomes

The primary outcome was a composite of a major cardiac complication (myocardial infarction or non-fatal cardiac arrest) or all-cause death within 30 days. This definition reflects elements used in prior large perioperative cohort studies, including the METS and Vascular Events in Noncardiac Surgery Patients Cohort Evaluation (VISION) studies,^3^^,^^16^ and was selected to provide a pragmatic and clinically relevant outcome measure. The secondary outcome was a postoperative in-hospital major complication, defined in alignment with recommendations from the joint task force of the European Society of Anaesthesiology and the European Society of Intensive Care Medicine on perioperative outcome measures.^17^

### Analyses

We used descriptive statistics to summarise baseline characteristics and outcomes. To evaluate whether the DASI improved prognostic risk estimation beyond guideline-recommended predictors, we fitted two baseline hierarchical logistic regression models for the primary outcome, each with a random intercept for the source cohort (i.e., METS vs. FIT After Surgery) to account for between-study heterogeneity. Model 1 included age and the Revised Cardiac Risk Index (RCRI).^1^^,^^18^^,^^19^ For the RCRI, we applied a modified definition of diabetes mellitus (treatment with any diabetes medication rather than insulin only) and categorised patients as 0 points, 1 point, 2 points, or ≥3 points.^19^ We used the aggregate RCRI score rather than individual component variables to reflect how the index is applied in clinical practice and contemporary guidelines. Model 2 additionally included preoperative BNP or NT-proBNP concentration. Natriuretic peptide concentrations were classified as low risk (BNP <92 ng/L or NT-proBNP <200 ng/L) or non-low risk, consistent with prior data, expert recommendations, and guideline-based practice.^20^, ^21^, ^22^ All variables were entered simultaneously, after which the DASI was added to each baseline model. These models were prespecified to reflect contemporary guideline-recommended approaches to perioperative cardiac risk assessment, which are anchored to age, RCRI, and natriuretic peptide concentrations.^1^^,^^2^^,^^18^ Accordingly, the primary objective of the analysis was to evaluate whether addition of the DASI improved prognostic risk estimation within these existing frameworks, rather than to derive a de novo prediction model or solely evaluate movement across predefined risk categories. The models did not include additional variables reported in individual studies, such as American Society of Anesthesiologists Physical Status (ASA-PS), haemoglobin concentration, or estimated glomerular filtration rate (eGFR), because they are not consistently incorporated into guideline-based risk assessment algorithms.

We used restricted cubic splines to examine the functional form of continuous variables (age, DASI) and to determine whether transformation or categorisation was required.^23^ Linearity assumptions were assessed using two complementary approaches: visual inspection of plots of the predictor against the log odds of the outcome, and analysis of variance comparing models with restricted cubic spline terms versus models assuming linearity.^23^ Multicollinearity was assessed using the variance inflation factor. Discrimination was quantified by the c-index,^23^ with values < 0.60 indicating poor discrimination, values between 0.60 and 0.75 indicating possibly helpful discrimination, and values > 0.75 indicating clearly useful discrimination.^24^ Improvement in model fit after inclusion of the DASI was first tested with the likelihood ratio test. Where model fit improved, we then evaluated incremental prognostic value using several complementary performance metrics to provide a multifaceted assessment of discrimination, calibration, risk reclassification, and clinical utility: fraction of new predictive information,^25^ continuous net reclassification improvement (NRI) statistic,^26^ change in c-index, integrated discrimination improvement (IDI) index,^26^ calibration plots, and decision curve analysis.^27^ Decision curve analyses were performed across clinically plausible risk thresholds (5%–20%),^2^^,^^20^^,^^22^ corresponding to levels at which perioperative management strategies such as postoperative troponin surveillance or additional preoperative cardiac evaluation may be considered. As an additional exploratory analysis requested during peer review, we evaluated categorical NRI using predefined risk categories (<5%, 5% to <10%, ≥10%), corresponding to clinically relevant cardiac risk thresholds.

Where inclusion of the DASI improved the performance of Model 1 or Model 2, we calculated predicted probabilities of the primary outcome across combinations of the predictor variables (i.e., age, RCRI category, DASI score, preoperative BNP or NT-proBNP concentration). The predicted risk estimates were used to derive DASI thresholds associated with predicted risks exceeding clinically meaningful cut-offs (≥5% and ≥10%).^22^ These analyses were intended to contextualise how DASI scores relate to absolute perioperative cardiac risk across different clinical scenarios rather than define fixed patient-level treatment thresholds.

We fitted a third hierarchical logistic regression model (Model 3) to assess whether the DASI provided incremental prognostic information for the secondary outcome beyond readily assessed risk factors. The baseline model included age, sex, and surgery type, classified as high-risk (intra-peritoneal, thoracic, or supra-inguinal vascular),^19^ major joint replacement, or other. The DASI was then added. Assessment of multicollinearity, linearity assumptions, and incremental prognostic value followed the same approach as for Models 1 and 2.

As a sensitivity analysis, we re-estimated Model 2 using continuous log-transformed natriuretic peptide concentration on a harmonised NT-proBNP scale instead of the dichotomous natriuretic peptide variable. For participants with BNP measurements, BNP values were converted to NT-proBNP using a validated conversion equation to enable pooled continuous modelling.^28^

As a secondary analysis, we repeated all modelling approaches (Models 1 to 3), substituting the four-question (modified) Duke Activity Status Index (M-DASI-4Q) in place of the full DASI. The M-DASI-4Q (Supplementary Table S3, Appendix) was developed as a simpler alternative to the full 12-item questionnaire, retaining four core items to estimate exercise capacity.^29^^,^^30^ Analytic procedures and performance metrics were identical to the primary analyses.

Missing baseline or outcome data were handled with multiple imputation by chained equations, with 30 imputed datasets generated and estimates pooled using Rubin’s rules. Standard diagnostic assessments of the imputation procedure were performed, including convergence and distributional plausibility checks. The outcome variables were included in the imputation model but individuals with missing outcomes were excluded from the analysis.^31^ Using the *mice* package in R (via the *quickpred* function), predictors were selected for inclusion based on an absolute correlation ≥ 0.20. Predictor variables in the imputation model are listed in Supplementary Table S4 (Appendix). Analyses were performed using R (version 4.5.3). Statistical significance was defined as a two-tailed p < 0.05 without adjustment for multiple comparisons.^32^ The number of participants included in this analysis was determined by the sample size calculations of the METS and FIT After Surgery studies.^3^^,^^12^

### Role of the funding source

The funders had no role in study design, data collection, data analysis, data interpretation, or writing of the report. The corresponding author had full access to all the data and final responsibility for the decision to submit for publication.

## Results

### Participant characteristics

The pooled cohort included 3485 patients who underwent surgery (Supplementary Fig. S1, Appendix), with 30-day follow-up complete for 3469 (99.5%). Baseline characteristics are shown in Table 1. The median age was 70 years (inter-quartile range [IQR] 65–75), 42% were female, 63% were classified as ASA-PS class III or IV, and 19% had an RCRI score of ≥2 points. Approximately 26% (n = 811) had elevated preoperative BNP or NT-proBNP concentrations, with the centre-specific natriuretic peptide assay being NT-proBNP for 80% (n = 2816) of the cohort. Most patients underwent major intra-peritoneal, urologic, or gynaecologic procedures, and 45% had cancer surgery. Postoperative outcomes are shown in Table 2. Overall, 3.2% (n = 110) experienced a major cardiac complication and 19% (n = 647) experienced a major complication. By 30 days, 103 patients (3.0%) experienced myocardial infarction, 10 (0.3%) experienced non-fatal cardiac arrest, and 20 (0.6%) died. Overall, 3.6% (n = 126) experienced the primary outcome of a major cardiac complication or death. Components of the composite endpoint were not mutually exclusive (Supplementary Table S5, Appendix).

**Table 1:** Characteristics of pooled cohort, and stratified by the primary outcome (30-day major cardiac complication or death).

	Cohort (N = 3485)	30-Day Major Cardiac Complication or Death (n = 126)	Alive at 30 Days without Major Cardiac Complication (n = 3345)	Missing Outcome (n = 14)
Demographics				
Median age (IQR) – y	70 (65–75)	73 (68–79)	70 (65–75)	73.5 (69–75)
Female sex – no. (%)	1451 (42%)	52 (41%)	1395 (42%)	4 (29%)
Baseline assessments				
Median 12-item DASI (IQR)	36.7 (23.5–50.7)	24.2 (17.3–44.7)	37.1 (23.5–50.7)	33.1 (16.3–47.8)
Missing	20	1	17	2
M-DASI-4Q				
0 points	339 (9.8%)	21 (17%)	317 (9.5%)	1 (8.3%)
1 point	859 (25%)	42 (34%)	815 (24%)	2 (17%)
2 points	623 (18%)	24 (19%)	597 (18%)	2 (17%)
3 points	911 (26%)	20 (16%)	885 (27%)	6 (50%)
4 points	733 (21%)	18 (14%)	714 (21%)	1 (8.3%)
Missing	20	1	17	2
Smoking status – no. (%)				
Any smoking in prior year	507 (15%)	18 (14%)	486 (15%)	3 (21%)
Remote smoking history	1396 (40%)	59 (47%)	1332 (40%)	5 (36%)
Non-smoker	1582 (45%)	49 (39%)	1527 (46%)	6 (43%)
Comorbid disease – no. (%)				
Ischaemic heart disease	545 (16%)	34 (27%)	507 (15%)	4 (29%)
Heart failure	96 (2.8%)	11 (8.7%)	85 (2.5%)	0 (0%)
Atrial fibrillation	267 (7.7%)	20 (16%)	247 (7.4%)	0 (0%)
Cerebrovascular disease	193 (5.5%)	12 (9.5%)	181 (5.4%)	0 (0%)
Peripheral artery disease	183 (5.3%)	12 (9.5%)	169 (5.1%)	2 (14%)
Diabetes mellitus	648 (19%)	31 (25%)	614 (18%)	3 (21%)
Hypertension	2023 (58%)	79 (63%)	1933 (58%)	11 (79%)
Renal replacement therapy	39 (1.1%)	1 (0.8%)	38 (1.1%)	0 (0%)
Preoperative renal insufficiency^a^	78 (2.4%)	6 (5.0%)	72 (2.3%)	0 (0%)
Missing	178	5	172	1
Obstructive pulmonary disease	515 (15%)	18 (14%)	493 (15%)	4 (29%)
Cancer				
Unrelated to planned surgery	220 (6.3%)	4 (3.2%)	215 (6.4%)	1 (7.1%)
Planned cancer surgery	1570 (45%)	58 (46%)	1506 (45%)	6 (43%)
Arthritis	1324 (38%)	49 (39%)	1273 (38%)	2 (14%)
ASA-PS class				
I	113 (3.3%)	0 (0%)	113 (3.4%)	0 (0%)
II	1158 (33%)	27 (22%)	1128 (34%)	3 (21%)
III	1755 (51%)	65 (52%)	1682 (50%)	8 (57%)
IV	445 (13%)	33 (26%)	409 (12%)	3 (21%)
Missing	14	1	13	0
Revised Cardiac Risk Index				
0 points	1206 (36%)	34 (28%)	1168 (37%)	4 (31%)
1 point	1444 (44%)	45 (37%)	1393 (44%)	6 (46%)
2 points	491 (15%)	26 (21%)	463 (15%)	2 (15%)
≥3 points	166 (5.0%)	16 (13%)	149 (4.7%)	1 (7.7%)
Missing	178	5	172	1
Elevated natriuretic peptide^b^	811 (26%)	57 (50%)	753 (25%)	1 (8.3%)
Missing	322	13	307	2
**Operative characteristics**				
Surgery type – no. (%)				
Vascular	140 (4.0%)	9 (7.1%)	131 (3.9%)	0 (0%)
Intra-thoracic	181 (5.2%)	3 (2.4%)	176 (5.3%)	2 (14%)
Intra-peritoneal	1319 (38%)	55 (44%)	1259 (38%)	5 (36%)
Major spine	390 (11%)	22 (17%)	364 (11%)	4 (29%)
Orthopaedic	371 (11%)	12 (9.5%)	358 (11%)	1 (7.1%)
Urologic or gynaecologic	853 (24%)	20 (16%)	831 (25%)	2 (14%)
Head-and-neck	176 (5.1%)	4 (3.2%)	172 (5.1%)	0 (0%)
Other	55 (1.6%)	1 (0.8%)	54 (1.6%)	0 (0%)

ASA-PS, American Society of Anesthesiologists Physical Status; DASI, Duke Activity Status Index; IQR, inter-quartile range; M-DASI-4Q, four-question (modified) Duke Activity Status Index.

a Defined as preoperative creatinine concentration ≥177 μmol/L or dialysis-dependence.

b Defined as B-type natriuretic peptide [BNP] ≥92 ng/L or N-terminal pro-B-type natriuretic peptide [NT-proBNP] ≥200 ng/L.

**Table 2:** Postoperative events during index hospitalisation.

	Overall cohort (N = 3485)^a^
Unexpected critical care admission – no. (%)	168 (4.8%)
Myocardial injury – no. (%)	886 (25%)
Major cardiac complication – no. (%)	110 (3.2%)
Non-fatal cardiac arrest – no. (%)	10 (0.3%)
Myocardial infarction – no. (%)	103 (3.0%)
Acute heart failure – no. (%)	10 (0.3%)
Venous thromboembolism – no. (%)	25 (0.7%)
Stroke or transient ischaemic attack – no. (%)	5 (0.1%)
Respiratory failure – no. (%)	66 (1.9%)
Pneumonia – no. (%)	65 (1.9%)
Surgical site infection – no. (%)	114 (3.3%)
New requirement for dialysis – no. (%)	14 (0.4%)
Re-operation – no. (%)	84 (2.4%)
Complication severity – no. (%)	
None	2464 (71%)
Mild	372 (11%)
Moderate	491 (14%)
Severe	135 (3.9%)
Fatal	21 (0.6%)
Major complication – n. (%)^b^	647 (19%)

IQR, inter-quartile range.

a One participant withdrew immediately after surgery, precluding capture of all subsequent in-hospital events, postoperative length-of-stay was missing for 1 additional patient, status at hospital discharge was missing for 4 additional patients, and complication severity was missing for 1 additional patient.

b Defined as a moderate, severe, or fatal complication.

Bivariate comparisons stratified by the primary and secondary outcomes are presented in Table 1 and Supplementary Table S6 (Appendix). In general, patients who experienced 30-day major cardiac complications or death were older, had lower DASI scores, were more frequently classified as high risk based on both the RCRI and ASA-PS, and had a greater burden of comorbidities (e.g., coronary artery disease, heart failure, atrial fibrillation, cerebrovascular disease, peripheral artery disease, diabetes, renal insufficiency). They also had higher preoperative natriuretic peptide concentrations.

### Prediction of 30-day postoperative major cardiac complications or death

Regression diagnostics showed no evidence of meaningful multicollinearity or violation of linearity assumptions (Supplementary Fig. S2, Appendix) for continuous predictors (age, DASI). DASI scores provided incremental prognostic value over clinical risk factors alone (Model 1), and beyond clinical factors plus natriuretic peptide concentration (Model 2), as reflected by the model log-likelihood, fraction of new predictive information, continuous NRI statistic, and adjusted odds ratio (Table 3). Additional categorical NRI analyses using predefined risk categories demonstrated minimal net risk reclassification with inclusion of the DASI (Table 3). Incorporation of DASI into the prediction models resulted in c-index values of 0.70 to 0.71, consistent with possibly useful discrimination. Parameter estimates for Models 1 and 2 are presented in Supplementary Table S7 (Appendix). Calibration plots demonstrated generally good agreement between predicted and observed risks across the observed range of predicted probabilities for both Model 1 and Model 2 (Supplementary Figs. S3 and S4, Appendix). Relatively few patients had predicted risks above 10%, resulting in greater uncertainty in calibration estimates in this upper range. For both Model 1 and Model 2, the baseline models demonstrated net clinical benefit across low-to-moderate decision thresholds (Supplementary Figs. S5 and S6, Appendix), ranging from approximately 5%–15%. Decision curves for models including the DASI showed substantial overlap with the base models, indicating that any net clinical benefit associated with DASI was small and inconsistent.

**Table 3:** Incremental predictive performance of DASI with respect to primary and secondary outcomes.

Covariates in model	aOR per 5-unit decrease in DASI (95% CI)	c-index	LRT	Fraction of new predictive information	IDI	Continuous net reclassification improvement index^a^		
						Events	Non-events	Overall (95% CI)
Primary outcome: 30-day major cardiac complication or death								
Model 1								
Age, RCRI		0.68 (0.63–0.73)						
+ DASI	1.11 (1.04–1.18)	0.70^b^ (0.65–0.74)	p < 0.001	17.9%	0.003 (0–0.006)	0.17 (0–0.35)	0.13 (0.10–0.16)	0.31 (0.13–0.48)
Model 2								
Age, RCRI, NP status^c^		0.70 (0.65–0.74)						
+ DASI	1.09 (1.02–1.16)	0.71^d^ (0.66–0.76)	p = 0.009	10.6%	0.001 (−0.001 to 0.006)	0.17 (0–0.35)	0.11 (0.07–0.14)	0.28 (0.10–0.46)
Sensitivity analysis								
Age, RCRI, log(NP)^e^		0.71 (0.66–0.76)						
+ DASI	1.09 (1.02–1.16)	0.72^f^ (0.68–0.77)	p = 0.01	9.6%	0.001 (−0.001 to 0.004)	0.15 (−0.03 to 0.32)	0.12 (0.08–0.15)	0.27 (0.09–0.44)
Secondary outcome: all-cause major postoperative complication								
Model 3								
Age, sex, surgery type		0.67 (0.65–0.69)						
+ DASI	1.07 (1.04–1.10)	0.68^g^ (0.66–0.70)	p < 0.001	7.3%	0.007 (0.003–0.01)	0.03 (−0.05 to 0.10)	0.11 (0.07–0.15)	0.14 (0.05–0.22)

aOR, adjusted odds ratio; DASI, Duke Activity Status Index; IDI, Integrated Discrimination Index; LRT, likelihood ratio test; NP, natriuretic peptide; NRI, net reclassification improvement; RCRI, Revised Cardiac Risk Index.

a Additional categorical NRI analyses using predefined risk categories (<5%, 5% to <10%, ≥10%) demonstrated small and non-significant net reclassification with inclusion of DASI in Model 1 (overall categorical NRI 0.033, 95% CI −0.061 to 0.126; event NRI 0.064, 95% CI −0.029 to 0.157; non-event NRI −0.031, 95% CI −0.043 to −0.018) and Model 2 (overall categorical NRI 0.001, 95% CI, −0.069 to 0.071; event NRI −0.002, 95% CI −0.074 to 0.071; non-event NRI 0.003, 95% CI −0.01 to 0.015).

b Difference in c-index was 0.018 (95% CI, −0.011 to 0.047; p = 0.23).

c Defined as B-type natriuretic peptide [BNP] ≥92 ng/L or N-terminal pro-B-type natriuretic peptide [NT-proBNP] ≥200 ng/L.

d Difference in c-index was 0.015 (95% CI, −0.005 to 0.035; p = 0.15).

e Natriuretic peptide concentration was modelled as a continuous variable using a harmonised, log-transformed NT-proBNP scale. For participants with BNP measurements, values were converted to NT-proBNP using a validated conversion equation.^28^

f Difference in c-index was 0.012 (95% CI, −0.006 to 0.03; p = 0.20).

g Difference in c-index was 0.009 (95% CI, 0–0.018; p = 0.04).

**Fig. 1:**
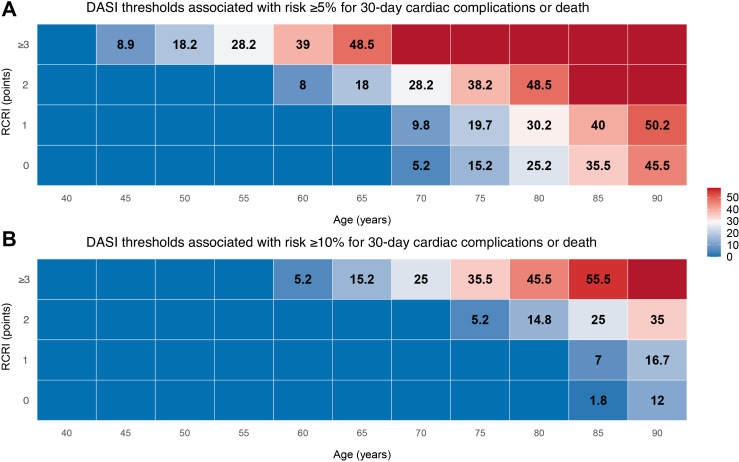
Predicted DASI thresholds below which 30-day risk of major cardiac complications or death exceeds prespecified levels. Legend: Heat maps displaying Duke Activity Status Index (DASI) score thresholds below which the predicted 30-day risk of major cardiac complications or death exceeds 5% (Panel A) and 10% (Panel B), based on hierarchical logistic regression models including age, Revised Cardiac Risk Index (RCRI), and DASI. Values in each cell represent the DASI score below which a patient would be predicted to exceed the specified risk threshold for the corresponding combination of age (x-axis) and RCRI (y-axis). Lower DASI values indicate poorer functional capacity. Colour gradients reflect increasing DASI threshold values, with blue denoting lower functional capacity thresholds and red denoting higher thresholds. Cells shaded solid red without a numerical value indicate that all patients with that age and RCRI combination are predicted to exceed the risk threshold regardless of DASI score. Conversely, solid blue cells without a numerical value indicate that all such patients are predicted to remain below the risk threshold irrespective of DASI score. For example, an 80-year-old patient with an RCRI score of ≥3 points is predicted to exceed a 5% risk regardless of DASI score, whereas a 70-year-old patient with an RCRI score of 2 points exceeds a 5% risk only when the DASI score is below approximately 28.

**Fig. 2:**
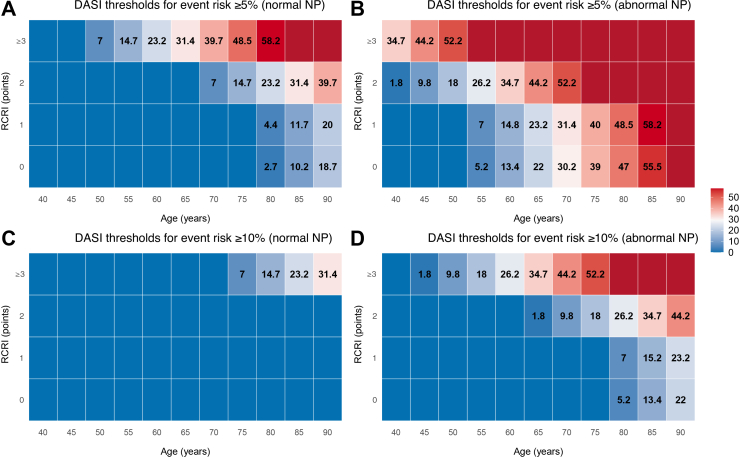
Predicted DASI thresholds below which 30-day risk of major cardiac complications or death exceeds prespecified levels, stratified by natriuretic peptide (BNP or NT-proBNP) status. Legend: Heat maps displaying Duke Activity Status Index (DASI) score thresholds below which the predicted 30-day risk of major cardiac complications or death exceeds 5% (Panels A and B) and 10% (Panels C and D), stratified by natriuretic peptide status, based on hierarchical logistic regression models including age, Revised Cardiac Risk Index (RCRI), DASI, and natriuretic peptide concentration. Natriuretic peptide status was classified as low risk or non-low risk (defined as B-type natriuretic peptide [BNP] ≥92 ng/L or N-terminal pro-B-type natriuretic peptide [NT-proBNP] ≥200 ng/L).^21^ Values within each cell represent the DASI score below which a patient would be predicted to exceed the specified risk threshold for the given combination of age (x-axis), RCRI (y-axis), and natriuretic peptide status. Lower DASI values indicate poorer functional capacity. Colour gradients reflect increasing DASI threshold values, with blue denoting lower thresholds and red denoting higher thresholds. Cells shaded solid red without a numerical value indicate that all patients within that stratum are predicted to exceed the risk threshold regardless of DASI score. Conversely, solid blue cells without a numerical value indicate that all such patients are predicted to remain below the risk threshold irrespective of DASI score. Across age and RCRI strata, elevated natriuretic peptide concentrations were associated with higher predicted absolute risk across a broader range of DASI scores compared with low-risk natriuretic peptide concentrations.

DASI score thresholds corresponding to predicted risks exceeding 5% and 10% for the primary outcome are shown in Figs. 1 and 2. Predicted risks associated with a given DASI score varied substantially according to age, RCRI, and natriuretic peptide status, consistent with interpretation of the DASI as a continuous prognostic marker whose implications depend on clinical context. For example, in the absence of natriuretic peptide testing (Fig. 1), a 70-year-old patient with an RCRI of 1 point exceeded a 5% predicted 30-day cardiac risk only when the DASI score was below approximately 10. In contrast, when natriuretic peptide concentrations were elevated (Fig. 2), the same patient exceeded the 5% risk threshold across a much broader range of DASI scores, including values up to approximately 31. Similarly, an 80-year-old patient with an RCRI of ≥3 points exceeded a 5% predicted risk regardless of DASI score, particularly in the presence of elevated natriuretic peptide concentrations.

Findings were materially unchanged in a sensitivity analysis in which Model 2 was re-estimated using continuous log-transformed natriuretic peptide concentration on a harmonised NT-proBNP scale. In this analysis, inclusion of DASI continued to improve overall model fit (likelihood ratio test p = 0.01), and the adjusted association between DASI and the primary outcome remained similar to the primary analysis (Supplementary Table S7, Appendix).

### Prediction of major postoperative complications

Regression diagnostics showed no evidence of meaningful multicollinearity or non-linearity for age and DASI (Supplementary Fig. S7, Appendix). DASI scores provided incremental prognostic value over clinical factors (Model 3), as demonstrated by the model log-likelihood, fraction of new predictive information, continuous NRI statistic, IDI statistic, and adjusted odds ratio (Table 3). Incorporation of DASI into the model resulted in possibly useful discrimination (c-index 0.68). Parameter estimates for Model 3 are presented in Supplementary Table S7 (Appendix). The baseline model demonstrated net clinical benefit across low-to-moderate decision thresholds, ranging from approximately 5%–25% (Supplementary Fig. S8, Appendix). Inclusion of the DASI yielded slightly higher net benefit across portions of this range; however, absolute differences were small, indicating only modest incremental clinical utility.

### Secondary analyses

The four-item M-DASI-4Q provided incremental prognostic value over a model based on clinical factors alone (p = 0.04) for predicting the primary outcome (Supplementary Table S8, Appendix), but not when natriuretic peptide concentration was included (p = 0.13). For the secondary outcome, the M-DASI-4Q improved overall model fit (p = 0.001), but its inclusion worsened reclassification of events (continuous NRI for events: −0.14; 95% CI −0.22 to −0.07). Parameter estimates for the modified Models 1 and 3 incorporating the M-DASI-4Q are shown in Supplementary Table S9 (Appendix).

## Discussion

In this international pooled cohort analysis of adults undergoing elective major non-cardiac surgery, preoperative functional capacity as measured by the full 12-item DASI provided incremental prognostic information for the primary composite outcome of 30-day major cardiac complications or death beyond guideline-recommended risk factors,^1^^,^^2^^,^^18^ including age, a clinical index (RCRI),^33^ and cardiac biomarkers (BNP or NT-proBNP).^20^ The DASI also provided smaller but detectable incremental prognostic value for all-cause major postoperative complications. Adding the DASI informed risk stratification by identifying DASI score ranges corresponding to clinically relevant 30-day cardiac risk thresholds, which varied according to age, comorbidity burden, and natriuretic peptide status. In contrast, the simpler four-item M-DASI-4Q showed weaker and less consistent prognostic performance, with evidence of worsened event classification when predicting major postoperative complications.

Our findings provide multicentre evidence to inform how self-reported functional capacity should be incorporated into evidence-based cardiac risk assessment for major non-cardiac surgery. Consistent with our prespecified design, we evaluated DASI within guideline-recommended risk frameworks rather than more complex models that are not routinely used in clinical practice. In this context, our results help to reinterpret current clinical practice guideline recommendations that endorse use of the DASI for preoperative cardiac risk stratification.^1^^,^^2^ We show that the prognostic implications of a given DASI score are continuous and context-dependent, where the absolute cardiac risk associated with a given DASI score varied meaningfully according to age, RCRI, and natriuretic peptide status. These findings indicate that the DASI functions best as a continuous prognostic marker rather than as a dichotomous screening test based on a single universal threshold. The heat-map risk thresholds presented in Figs. 1 and 2 help operationalise this concept by illustrating how the same DASI score may correspond to very different predicted risks depending on the broader clinical context. Importantly, these figures illustrate model-based risk rather than precise patient-level estimates. This framework provides a more clinically coherent approach to integrating functional capacity assessment into perioperative decision-making than reliance on binary cut-points alone. Future decision-support tools may further facilitate clinical implementation by enabling more explicit representation of uncertainty in predicted risks, which is not easily conveyed in static visualisations such as heat maps. Overall, our findings establish how the DASI should be interpreted alongside established predictors in contemporary practice, with the results helping to refine, rather than contradict, current guideline recommendations. The DASI contains prognostic information not captured by traditional clinical indices, but it should be used to contextualise risk estimation rather than to independently dictate downstream testing or management.

The DASI thresholds presented in Figs. 1 and 2 were anchored to predicted cardiac event risks of ≥5% and ≥10%. Although alternative DASI thresholds could be generated for other absolute risk tolerances and healthcare settings, we reasoned a priori that these cut-points are relevant to perioperative practice. For example, a predicted risk of ≥5% identifies a population in whom routine postoperative troponin surveillance is likely beneficial. Given that only 32% of patients with postoperative myocardial infarction report ischaemic symptoms, routine testing in this group would help detect one otherwise missed myocardial infarction for every 30 troponin tests performed.^34^ Conversely, patients with predicted cardiac risks of ≥10% represent a group in whom additional preoperative cardiac testing, specialist consultation, or enhanced postoperative monitoring may be appropriate. However, relatively few patients occupied this higher-risk range, and estimates in this region were therefore less precise. Overall, framing functional capacity thresholds around clinically relevant absolute risk levels may help contextualise how structured functional capacity assessment is incorporated into perioperative decision-making.

Although the DASI also showed associations with all-cause major complications, its incremental prognostic performance was less compelling than for cardiac events. For example, when added to clinical factors, it contributed a smaller fraction of new predictive information (17.9% vs. 7.3%) and a smaller improvement in net reclassification (NRI 0.31 vs. 0.14). This pattern is biologically plausible and likely reflects the greater heterogeneity and multifactorial aetiology of all-cause major complications compared with cardiac events. Our findings also raise caution regarding simplified functional capacity instruments. The four-item M-DASI-4Q was developed to predict preoperative cardiopulmonary exercise testing performance,^29^ but demonstrated weaker and less consistent incremental prognostic performance than the full 12-item DASI for prediction of cardiac events. There was stronger statistical evidence of incremental prognostic value for prediction of all-cause major complications, with approximately 35% of patients with ≤1 M-DASI-4Q items exhibiting elevated complication risk. However, this gain occurred at the cost of worsened event reclassification. These findings suggest that the parsimony of the M-DASI-4Q may come at the expense of clinically meaningful prognostic value. The full DASI therefore remains the preferable instrument when structured functional capacity assessment is used to inform preoperative risk assessment.

From a clinical perspective, our results support integration of the full 12-item DASI as a component of a multidimensional perioperative risk assessment framework that also incorporates age, comorbidity burden, and cardiac biomarkers. As shown in Figs. 1 and 2, the DASI is likely to be most useful in patients with intermediate to higher estimated perioperative cardiac risk based on age and RCRI, particularly when natriuretic peptide concentrations are unavailable or discordant with other clinical indicators. In such settings, structured functional capacity assessment may help refine decisions about postoperative troponin surveillance, additional preoperative testing, and shared decision-making. However, given the modest incremental prognostic value observed, the clinical impact of incorporating DASI into decision-making remains to be established.

Future research should therefore focus on how the DASI informs downstream perioperative management, including selection for preoperative cardiac imaging, intraoperative haemodynamic management, and enhanced postoperative surveillance. An important next step will be to determine whether DASI-guided clinical decision-making improves patient outcomes compared with existing approaches based on routinely available clinical predictors. In parallel, further work is needed to develop and validate more comprehensive and pragmatic prediction tools for preoperative cardiac risk assessment. Such tools should build on current guideline-recommended frameworks while systematically evaluating which predictors to include in a parsimonious and implementable model. Potential predictors include guideline-endorsed variables (e.g., DASI, RCRI, natriuretic peptides), other candidates identified in the literature (e.g., ASA-PS, haemoglobin concentration, eGFR), alternative structured functional capacity measures,^7^ and established clinical risk indices.^35^ These efforts may help define the role of structured functional capacity assessment within implementable decision support tools for clinical practice.

Our study has several strengths. It leverages two large rigorously conducted multicentre prospective cohorts with harmonised exposure assessment, routine postoperative biomarker surveillance, blinded outcome adjudication, and near-complete 30-day follow-up. The large sample enabled precise estimation of prognostic associations across a broad range of age, comorbidity burden, and functional capacity. Notably, the pooled cohort exceeded the minimum recommended sample size (n = 2592) required to derive a robust prediction model with the characteristics of Model 2 (c-index 0.71, event rate 3.6%, six model parameters),^36^ which reduces concerns about model instability and overfitting. These criteria were derived for the development of new prediction models and are therefore likely conservative in the context of incremental prognostic analyses. The analytic framework incorporated contemporary best practices for prognostic modelling, including hierarchical regression to account for clustering within individual cohorts, flexible modelling of continuous predictors, multiple imputation for missing data, and the use of complementary metrics to characterise incremental prognostic performance.^37^

Several limitations should also be noted. First, all prediction models demonstrated only possibly helpful discrimination (c-indices <0.75), which likely reflects the inherent difficulty of achieving strong discrimination using preoperative data alone. Meaningful improvements in discrimination will probably require incorporation of intraoperative and early postoperative factors, such as haemodynamic instability or bleeding events.^38^ Second, addition of the DASI resulted in only minimal changes in c-index, limited movement across clinically relevant risk categories, and did not demonstrate strong or consistent net clinical benefit on decision curve analysis. However, change in c-index is known to be statistically insensitive to meaningful improvements in risk estimation, and decision curve analysis addresses the clinical utility of using a model to guide management decisions rather than the narrower question of whether a predictor improves risk estimation.^37^ In addition, interpretation of categorical NRI may be influenced by the number and placement of risk categories, particularly when relatively few events occur within specific strata or when calibration is imperfect.^39^ Consistent with contemporary methodological guidance, we therefore incorporated several complementary methods for evaluating incremental prognostic value.^37^^,^^40^ Third, postoperative myocardial injury was not included as an outcome in this analysis, as it represents a broader and more biologically heterogeneous construct than clinically adjudicated myocardial infarction, and is not currently incorporated into established preoperative risk stratification frameworks. Fourth, preoperative natriuretic peptide concentrations were missing in approximately 9% of participants. Although missing data are an inherent challenge in prospective studies and the extent of missingness was within expected ranges, we addressed this limitation using multiple imputation, which permits valid and efficient inference under the missing-at-random assumption.^41^ Finally, the pooled cohort was drawn from high-income healthcare settings, and the generalisability of these findings to other surgical populations and health systems remains uncertain. The transportability of the estimated risk thresholds and the incremental prognostic value of the DASI should therefore be evaluated in external samples with different case-mix, perioperative practices, and baseline risk profiles.

In conclusion, this pooled cohort analysis found that the full 12-item DASI provided incremental prognostic information for estimating perioperative cardiac risk beyond guideline-recommended clinical predictors, including age, RCRI, and natriuretic peptide concentration. Refining current guideline recommendations, we found that the DASI functions most effectively as a continuous prognostic marker whose implications depend jointly on age, comorbidity burden, and natriuretic peptide status. However, the incremental improvement in predictive performance beyond existing clinical models was modest, and the clinical impact of incorporating DASI into perioperative decision-making remains to be established. Overall, these findings provide a more precise evidence-based framework for integrating structured self-reported functional capacity assessment into preoperative cardiac risk evaluation.

## Contributors

DNW and DIM conceived the study. DNW, KX, and DIM developed the methodology. DNW secured funding. DNW and KX curated the data. DNW, BHC, ED, TEFA, RMP, PSM, JFD, CD, LAA, AJ, BR and DIM contributed to investigation. DNW managed the project. DNW supervised the study. KX and DNW performed the formal analysis. DNW drafted the original manuscript. All authors critically revised the manuscript, approved the final version for submission, and agree to be accountable for the work. All authors had full access to all study data. DNW and KX verified the underlying data and take responsibility for its integrity and accuracy.

## Data sharing statement

This pooled analysis includes data from participating centres governed by national regulations that prohibit external sharing of individual-level participant information, even in de-identified form. Accordingly, de-identified data from the combined pooled dataset cannot be shared. To support transparency and reproducibility, we will make available the analytic code, detailed model specifications, and a structured variable dictionary sufficient to replicate the analyses using similar datasets. These materials will be provided upon reasonable request to the corresponding author.

## Declaration of interests

DNW and DIM are members of the Board of Directors of the International Anesthesia Research Society, unrelated to this work. DNW has served on advisory boards for Vertex Pharmaceuticals, unrelated to this work. ED reports receiving research grants for investigator-initiated studies from Roche Diagnostics, Abbott Laboratories, and Diagnoptics, and has served on advisory boards for Roche Diagnostics and Abbott Laboratories regarding perioperative use of biomarkers, all unrelated to this work. TEFA has received research funding from the National Institute for Health and Care Research (CL-2021-19-501), Barts Charity, the Academy of Medical Sciences, the Royal College of Anaesthetists, and the British Journal of Anaesthesia, and has received honoraria from Merck Sharp & Dohme, Edwards Lifesciences, and Elsevier; all unrelated to this work. All other authors declare no competing interests.
