# Supplementary material for: Prognostic value of the Duke Activity Status Index for preoperative cardiac risk stratification: an international pooled cohort study
Source: eClinicalMedicine. 2026 Jun 11;96:104015. doi: 10.1016/j.eclinm.2026.104015 (PMC13276150; doi:10.1016/j.eclinm.2026.104015)
Supplement: Supplementary Figs. S1–S8 and Tables S1–S9 [file mmc1.pdf]

WEB EXTRA MATERIAL: SUPPLEMENTARY APPENDIX

This web extra material has been provided to give readers additional information about this work.

WEB EXTRA MATERIAL TABLE OF CONTENTS

Table of Contents

List of METS Study Investigators 3

List of FIT After Surgery Study Investigators 4

Supplementary Methods 5

Overview of Study Design and Procedures in the METS Study 5

Overview of Study Design and Procedures in the FIT After Surgery Study 5

Figure S1: Pooled cohort assembly and follow-up 6

Figure S2: Association between continuous Duke Activity Status Index score and predicted log odds of the primary outcome, modelled using restricted cubic splines. 7

Figure S3: Pooled calibration plot for the primary outcome model including age, Revised Cardiac Risk Index, and DASI. 8

Figure S4: Pooled calibration plot for the primary outcome model including age, Revised Cardiac Risk Index, DASI, and natriuretic peptide status. 9

Figure S5: Net clinical benefit of adding DASI to age and Revised Cardiac Risk Index for predicting 30-day cardiac complications or death. 10

Figure S6: Net clinical benefit of adding DASI to age, Revised Cardiac Risk Index, and natriuretic peptide concentration (BNP or NT-proBNP) for predicting 30-day cardiac complications or death. 11

Figure S7: Association between continuous Duke Activity Status Index score and predicted log odds of the secondary outcome, modelled using restricted cubic splines 12

Figure S8: Net clinical benefit of adding DASI to age, sex, and surgery type for predicting major complications. 13

Table S1: Scoring scheme for the DASI 14

Table S2: Definitions of postoperative events 15

Table S3: Scoring scheme for the four-question (modified) DASI (M-DASI-4Q) 17

Table S4: Variables included in the multiple imputation model 18

Table S5: Component overlap within the 30-day primary composite outcome 19

Table S6: Bivariate comparisons between patients who did versus did not experience the secondary outcome (in-hospital major postoperative complication) 20

Table S7: Adjusted association of DASI with primary and secondary outcomes 22

Table S8: Additional predictive performance of M-DASI-4Q with respect to primary and secondary outcomes 23

Table S9: Adjusted association of M-DASI-4Q with primary and secondary outcomes 24

Appendix References 25

List of METS Study Investigators

1. **Study Site Investigators**

   Australia – *Alfred Hospital*: P S Myles (Site Co-Lead), M A Shulman (Site Co-Lead), S Wallace, C Farrington, B Thompson (Site CPET Lead), M Ellis, B Borg; *John Hunter Hospital*: R K Kerridge (Site Lead), J Douglas, J Brannan, J Pretto; *Nambour General Hospital*: M G Godsall (Site Co-Lead), N Beauchamp (Site Co-Lead), S Allen, A Kennedy, E Wright, J Malherbe; *Peter McCallum Cancer Centre*: H Ismail (Site Co-Lead), B Riedel (Site Co-Lead), A Melville, H Sivakumar, A Murmane, K Kenchington, Y Kirabiyik; *Prince Charles Hospital*: U Gurunathan (Site Lead), C Stonell, K Brunello, K Steele, O Tronstad, P Masel, A Dent, E Smith, A Bodger, M Abolfathi; *Princess Alexandra Hospital*: P Sivalingam (Site Co-Lead), A Hall (Site Co-Lead); *Royal Adelaide Hospital*: T W Painter (Site Co-Lead), S Macklin (Site Co-Lead), A Elliott, A M Carrera; *Royal Hobart Hospital*: N C S Terblanche (Site Lead); S Pitt, J Samuels, C Wilde; *Royal Melbourne Hospital*: K Leslie (Site Lead), A MacCormick; *Western Health*: D Bramley (Site Lead), A M Southcott, J Grant, H Taylor, S Bates, M Towns, A Tippett, F Marshall
   Canada – *St Michael's Hospital*: C D Mazer (Site Lead), J Kunasingam, A Yagnik, C Crescini, S Yagnik; *Sunnybrook Health Sciences Centre*: C J L McCartney (Site Co-Lead), S Choi (Site Co-Lead), P Somascanthan, K Flores; *Toronto General Hospital*: D N Wijeysundera (Site Co-Lead), W S Beattie (Site Co-Lead), K Karkouti, H A Clarke, A Jerath, S A McCluskey, M Wasowicz, J T Granton (Site CPET Lead), L Day, J Pazmino-Canizares; *Toronto Rehabilitation Institute – Rumsey Centre*: P Oh (Site Lead), R Belliard, L Lee, K Dobson; *Toronto Western Hospital*: V Chan (Site Lead), R Brull, N Ami, M Stanbrook (Site CPET Lead)
   New Zealand - *Auckland City Hospital*: K Hagen (Site Lead), D Campbell, T Short, J Van Der Westhuizen, K Higgie, H Lindsay, R Jang, C Wong, D Mcallister, M Ali, J Kumar, E Waymouth, C Kim; *Middlemore Hospital*: J Dimech (Site Co-Lead), M Lorimer (Site Co-Lead), J Tai, R Miller, R Sara, A Collingwood, S Olliff, S Gabriel, H Houston; *Wellington Hospital*: P Dalley (Site Lead), S Hurford, A Hunt, L Andrews, L Navarra, A Jason-Smith, H Thompson, N McMillan, G Back
   United Kingdom – *Aberdeen Royal Infirmary*: B L Croal (Site Lead), M Lum; *Royal Free Hospital*: D Martin (Site Lead), S James, H Filipe, M Pinto, S Kynaston; *Royal London Hospital*: R M Pearse (Site Lead), T E F Abbott, M Phull, C Beilstein, P Bodger, K Everingham, Y Hu, E Niebrzegowska, C Corriea, T Creary, M Januszewska, T Ahmad, J Whalley, R Haslop, J McNeil, A Brown, N MacDonald, M Pakats, K Greaves; *Royal Marsden Hospital*: S Jhanji (Site Co-Lead), R Raobaikady (Site Co-Lead), E Black, M Rooms, H Lawrence, M Koutra, K Pirie, M Gertsman; *Southampton General Hospital*: S Jack (Site Co-Lead), M Celinski (Site Co-Lead), D Levett, M Edwards, K Salmon, C Bolger, L Loughney, L Seaward, H Collins, B Tyrell, N Tantony, K Golder; *University College London Hospital*: G L Ackland (Site Lead), R C M Stephens, L Gallego-Paredes, A Reyes, A Gutierrez del Arroyo; *Whipps Cross Hospital*: A Raj (Site Lead), R Lifford

2. **International and National Coordinators** – B H Cuthbertson (International Co-Principal Investigator), D N Wijeysundera (International Co-Principal Investigator), R M Pearse, P S Myles, T E F Abbott, M A Shulman

3. **Central Project Office Operations Committee** – B H Cuthbertson, D N Wijeysundera, E Torres, A Ambosta, M Melo, M Mamdani, K E Thorpe, R M Pearse, T E F Abbott, P S Myles, M A Shulman, S Wallace, C Farrington, B L Croal

4. **Cardiopulmonary Exercise Testing Methods Committee** – M P W Grocott, J T Granton, P Oh, B Thompson, D Levett

5. **Outcome Adjudication Committee** – G Hillis (Chair), W S Beattie, H C Wijeysundera

6. **International Steering Committee –** B H Cuthbertson (International Co-Principal Investigator), D N Wijeysundera (International Co-Principal Investigator), R M Pearse, M A Shulman, T E F Abbott, E Torres, A Ambosta, B L Croal, J T Granton, K E Thorpe, M P W Grocott, C Farrington, S Wallace, P S Myles

List of FIT After Surgery Study Investigators

1. **<u>Study Site Investigators</u>:**
    a. *St. Michael's Hospital – Unity Health Toronto* (Toronto, Ontario): D N Wijeysundera (Site Lead), KS Ladha (Site Co-Lead), CD Mazer, J F Daza, C Diep, J Pazmino-Canizares, G Mattina, S Drozdz, R Nayar, C Hanley, L A Amado, J F Morales, S Gandotra, A Goel, S M Pereira, T R Chesney, M Louridas, C Wong, S Lee, N Rajapakse
    b. *The Ottawa Hospital – Ottawa Civic and Ottawa General Sites* (Ottawa, Ontario): D I McIsaac (Site Lead), R H Breau (Site Co-Lead), M Lalu (Site Co-Lead), S Abdellatif, S Gagne, E Hladkowicz, J Hutton
    c. *Centre Hospitalier de l'Université de Montréal* (Montreal, Quebec): E Duceppe (Site Lead), I Chergui, MF Boko, L M Drudi, C Richard, F Saad, F Vandenbroucke-Menu
    d. *Foothills Medical Centre* (Calgary, Alberta): M Davis (Site Lead), K Zarnke (Site Co-Lead), M Bosch (Site Co-Lead)
    e. *Humber River Hospital – Humber River Health* (Toronto, Ontario): S Avramescu (Site Lead), T Kerelska (Site Co-Lead), S Charummoottil
    f. *Juravinski Hospital – Hamilton Health Sciences* (Hamilton, Ontario): P E Serrano (Site Lead), L Ruo, Y Essaji
    g. *Kingston Health Sciences Centre* (Kingston, Ontario): J Van Vlymen (Site Lead), D Dumerton, R Tod, B Smethurst, E Vowotor, J Dion, M Karizhenskaia, A Malik, S Jain
    h. *Mississauga Hospital – Trillium Health Partners* (Mississauga, Ontario): H El Beheiry (Site Lead)
    i. *Mount Sinai Hospital – Sinai Health* (Toronto, Ontario): N Siddiqui (Site Lead), E Kennedy, S Khandadashpoor.
    j. *Queen Elizabeth II Health Sciences Centre* (Halifax, Nova Scotia): D MacDonald (Site Lead), R Spence, F Bonazza, A Zahavich
    k. *St. Boniface Hospital* (Winnipeg, Manitoba): E Jacobsohn (Site Lead), M Thorleifson (Site Co-Lead), S L Russell (Site Co-Lead), H Bagry (Site Co-Lead)
    l. *St. Paul's Hospital – Providence Health Care* (Vancouver, British Columbia): S Y MacDonell (Site Lead), V Lyon, T Barnes, J Dale-Gandar, N Y Edwards
    m. *Sunnybrook Health Sciences Centre* (Toronto, Ontario): S Choi (Site Lead), A Jerath, L Kaustov, A Fleet, S Shaheen
    n. *Toronto General Hospital – University Health Network* (Toronto, Ontario): M Parotto (Site Lead), S A McCluskey (Site Co-Lead), H Poonawala
    o. *Toronto Western Hospital – University Health Network* (Toronto, Ontario): G Lorello (Site Lead), E Al Azazi
    p. *University of Alberta Hospital* (Edmonton, Alberta): D Dillane (Site Lead), J Green (Site Co-Lead), M Alsaif

2. **<u>Operations Committee</u>:** D N Wijeysundera (Principal Investigator), D I McIsaac, S Ehtesham, E Hladkowicz, K S Ladha, J Pazmino-Canizares, J F Daza, C Diep, G Mattina, M Tessier

3. **<u>Outcome Adjudication Committee</u>:** D N Wijeysundera, J F Daza, C Diep

4. **<u>Steering Committee</u>:** D N Wijeysundera (Principal Investigator), D I McIsaac, S M H Alibhai, K S Ladha, C D Mazer, M Puts, T R Chesney, A C Wei, S Ehtesham

5. **<u>Central Project Office</u>:** S Ehtesham, S Nnorom, N F Syeda, A Ferenc, K Xu, G Lebovic, C Keown-Stoneman, C King, M Yang, K Kuang, J Wu

Supplementary Methods

Overview of Study Design and Procedures in the METS Study

Participants were adults aged ≥40 years who were scheduled for elective non-cardiac surgery under general or regional anaesthesia, with an anticipated postoperative hospital stay of at least one night. Eligible patients were required to have ≥1 risk factors for coronary artery disease or cardiac complications. These risk factors included coronary artery disease, heart failure, cerebrovascular disease, diabetes mellitus requiring pharmacological treatment, preoperative renal insufficiency (dialysis or estimated glomerular filtration rate ≤60 mL/min/1·73 m^2), peripheral artery disease, hypertension, current or recent smoking (any smoking within year preceding surgery), age ≥70 years, or high-risk surgery (intra-peritoneal, intra-thoracic, supra-inguinal vascular, or lower-extremity vascular procedures). Exclusion criteria included endovascular procedures, presence of an automated implantable cardioverter-defibrillator, and absolute contraindications to cardiopulmonary exercise testing.[1] Participants completed the Duke Activity Status Index (DASI) questionnaire at the time of study enrolment.[2] Blood samples were obtained at any point between recruitment and surgery to measure N-terminal pro-B-type natriuretic peptide (NT-proBNP) concentrations. Samples were stored at -70°C to -80°C at each participating site and subsequently analysed centrally at Aberdeen Royal Infirmary (Aberdeen, UK) using the Siemens Vista™ immunoassay analyser (Siemens Healthcare Diagnostics Ltd, Frimley, UK). After surgery, participants underwent daily 12-lead electrocardiography and blood sampling for measurement of cardiac troponin concentrations until postoperative day three or hospital discharge, whichever occurred first. Each site used its locally available troponin assay. Myocardial injury and myocardial infarction were adjudicated by an independent committee blinded to DASI and NT-proBNP results.[3] Research personnel assessed participants daily during the index hospital admission to ascertain postoperative complications. The most severe complication experienced by each participant was classified as mild, moderate, severe, or fatal using a modified International Surgical Outcomes Study (ISOS) severity classification.[4] After discharge, participants were contacted at 30 days to determine vital status and ascertain intervening medical events.

Overview of Study Design and Procedures in the FIT After Surgery Study

Participants were adults aged ≥65 years who were scheduled for elective non-cardiac surgery with an anticipated postoperative hospital stay of at least two nights. Elective surgery included time-sensitive scheduled procedures, such as cancer surgery performed with curative intent. Exclusion criteria included endovascular procedures, major joint replacement surgery, intracranial neurosurgery, palliative surgery, and documented severe dementia.[5] At enrolment, participants completed the DASI questionnaire. Blood samples were obtained to measure preoperative natriuretic peptide concentrations, either B-type natriuretic peptide (BNP) or NT-proBNP, depending on local laboratory availability. Following surgery, participants underwent daily blood sampling for measurement of cardiac troponin concentrations until postoperative day three or hospital discharge, whichever occurred first. Each site used its locally available troponin assay. Patients with elevated troponin also underwent 12-lead electrocardiography. Myocardial injury and myocardial infarction were adjudicated by an independent outcomes committee blinded to DASI and natriuretic peptide results.[6] Other postoperative complications were assessed daily during the index hospital admission. The most severe complication experienced by each participant was classified as mild, moderate, severe, or fatal using the same modified ISOS classification. After discharge, participants were contacted 30 days after surgery to determine vital status and ascertain intervening complications.

Figure S1: Pooled cohort assembly and follow-up

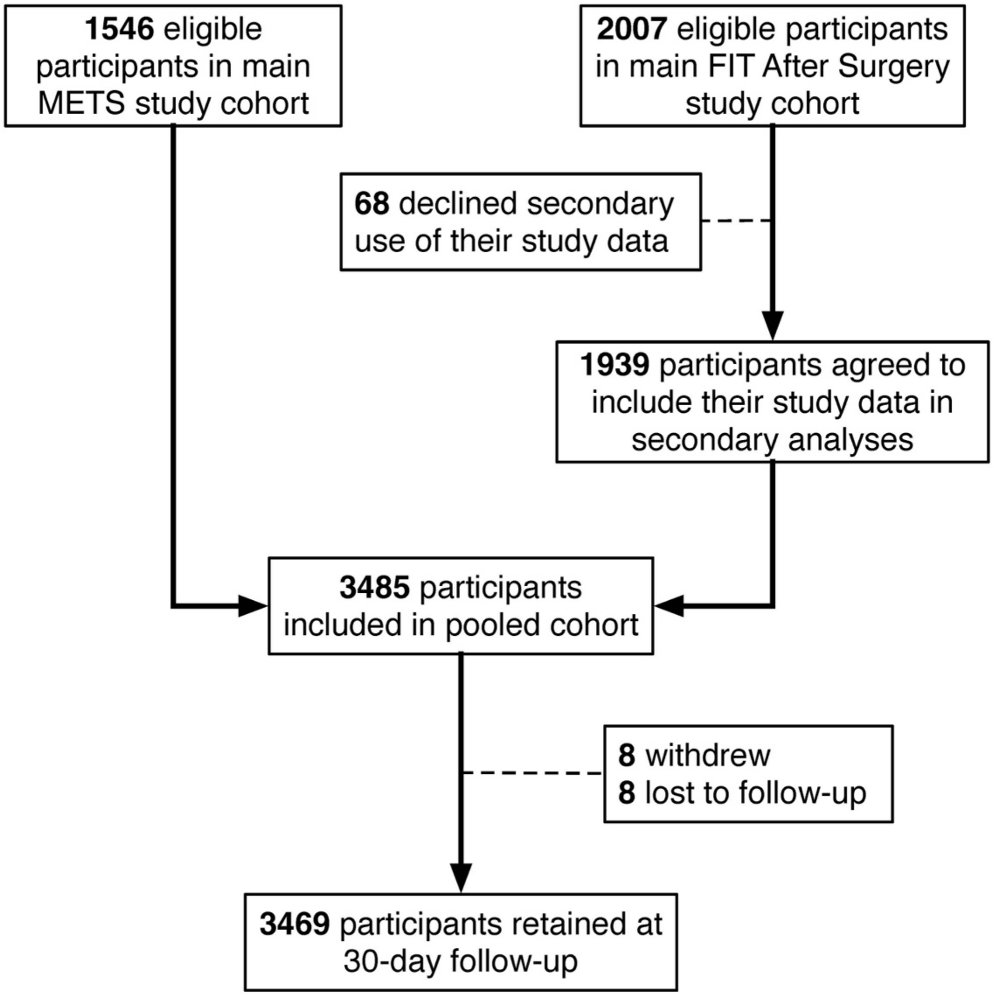

Figure S2: Association between continuous Duke Activity Status Index score and predicted log odds of the primary outcome, modelled using restricted cubic splines.

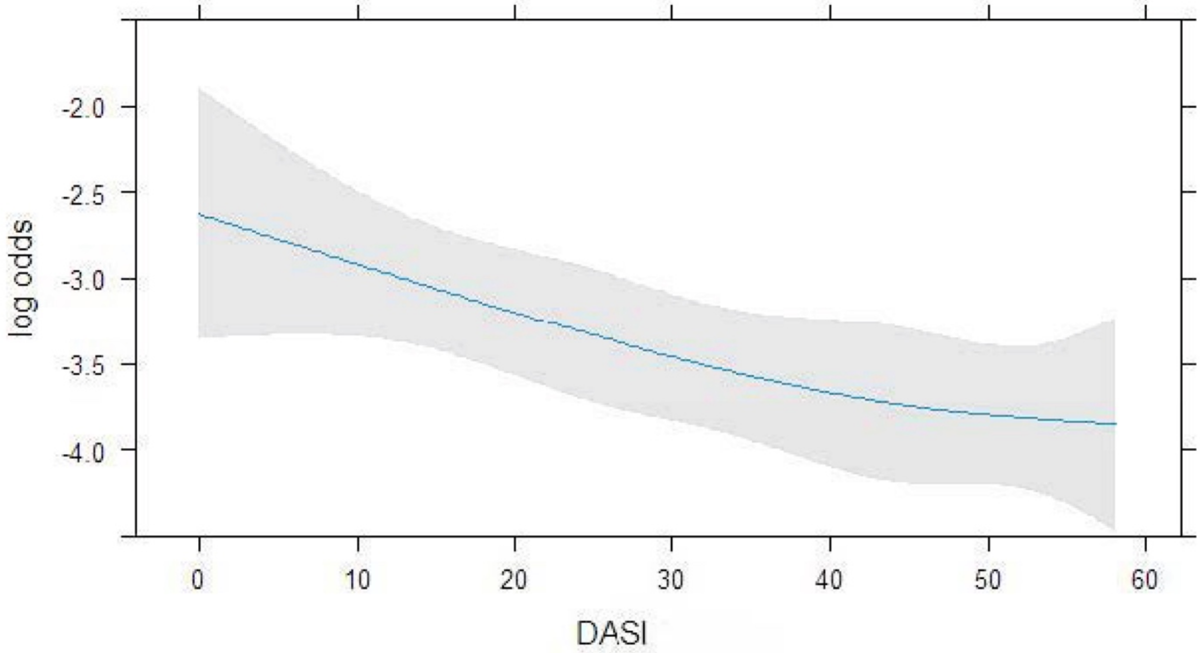

Legend: Predicted log odds of the primary outcome (30-day major cardiac complication or death) were derived from a logistic regression model including age, Revised Cardiac Risk Index, and DASI modelled using restricted cubic splines with four knots. Knots were placed at equally spaced quantiles between 0·05 and 0·95 (i.e., 5th, 35th, 65th, and 95th percentiles). The shaded area represents 95% confidence intervals. There was no evidence of significant nonlinearity for DASI (p=0·77 for nonlinear terms)

Figure S3: Pooled calibration plot for the primary outcome model including age, Revised Cardiac Risk Index, and DASI.

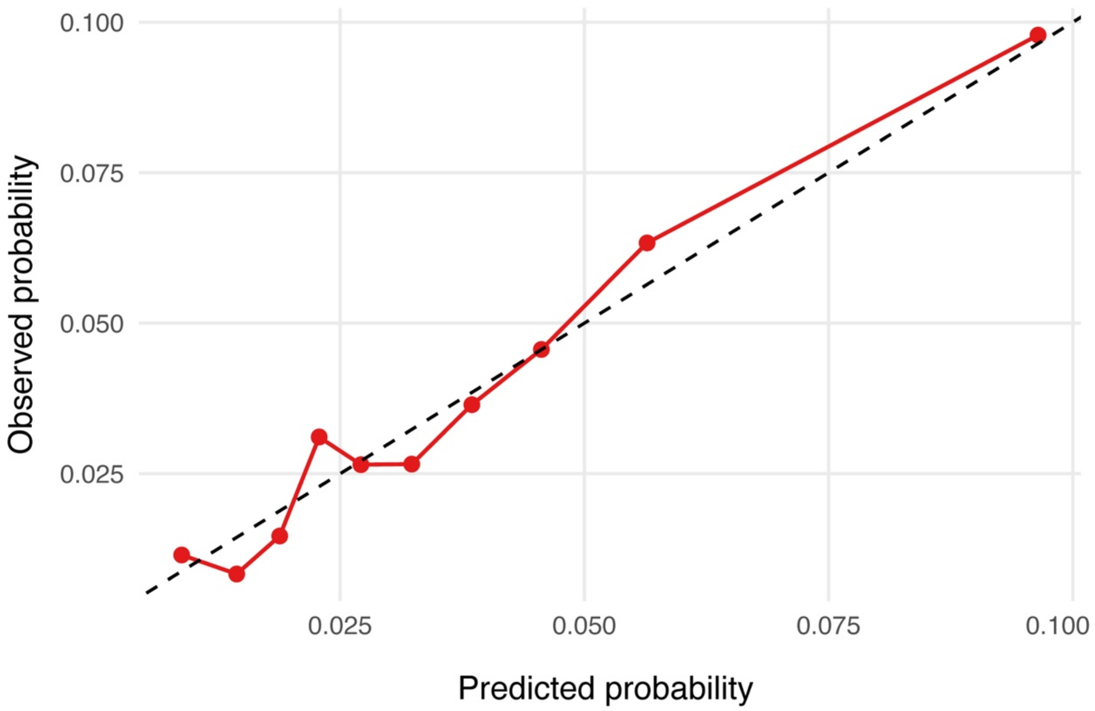

Legend: Observed versus predicted probabilities of the primary outcome (30-day major cardiac complication or death) for Model 1. Calibration was assessed by pooling predictions across imputed datasets and comparing observed event rates with mean predicted probabilities within deciles of predicted risk. Points represent grouped observed event rates plotted against mean predicted probabilities, and the dashed line indicates perfect calibration. Overall, calibration was acceptable across the observed prediction range. The pooled calibration intercept was 0·05 (95% CI, -0·78-0·87), the pooled calibration slope was 1·02 (95% CI, 0·75-1·28), and the pooled Brier score was 0·0342.

.

Figure S4: Pooled calibration plot for the primary outcome model including age, Revised Cardiac Risk Index, DASI, and natriuretic peptide status.

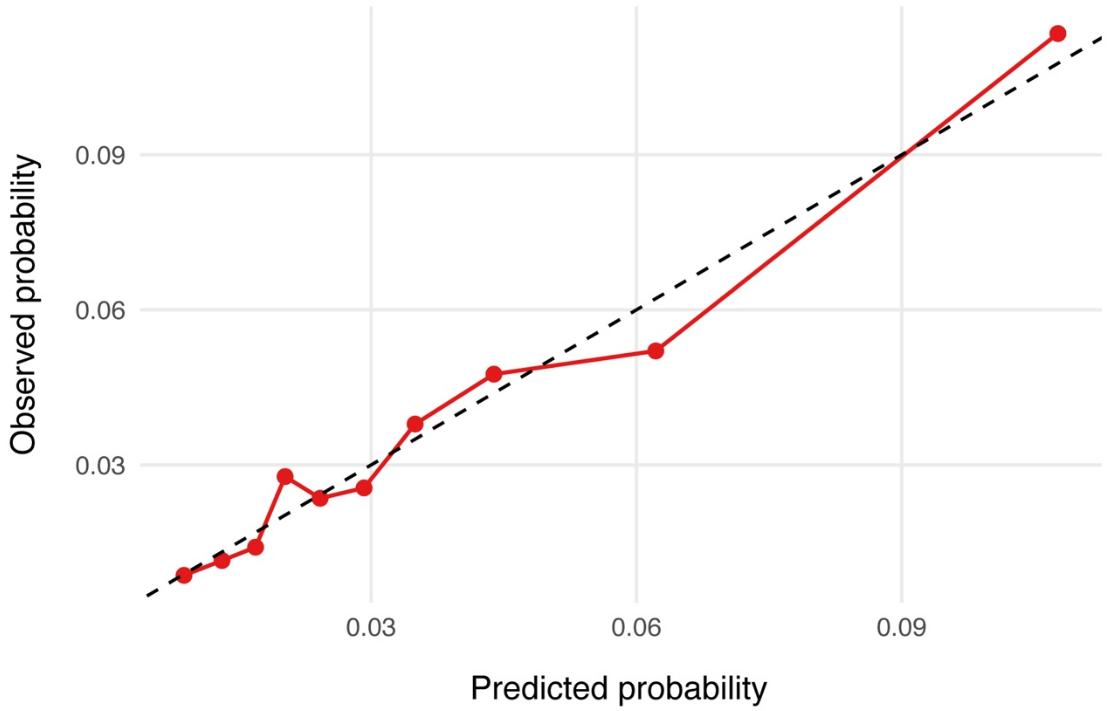

Legend: Observed versus predicted probabilities of the primary outcome (30-day major cardiac complication or death) for Model 2. Calibration was assessed by pooling predictions across imputed datasets and comparing observed event rates with mean predicted probabilities within deciles of predicted risk. Points represent grouped observed event rates plotted against mean predicted probabilities, and the dashed line indicates perfect calibration. Overall, calibration was acceptable across the observed prediction range. The pooled calibration intercept (95% CI) was 0·05 (-0·67-0·78), the pooled calibration slope was 1·02 (95% CI, 0·78-1·25) and the pooled Brier score was 0·0339.

Figure S5: Net clinical benefit of adding DASI to age and Revised Cardiac Risk Index for predicting 30-day cardiac complications or death.

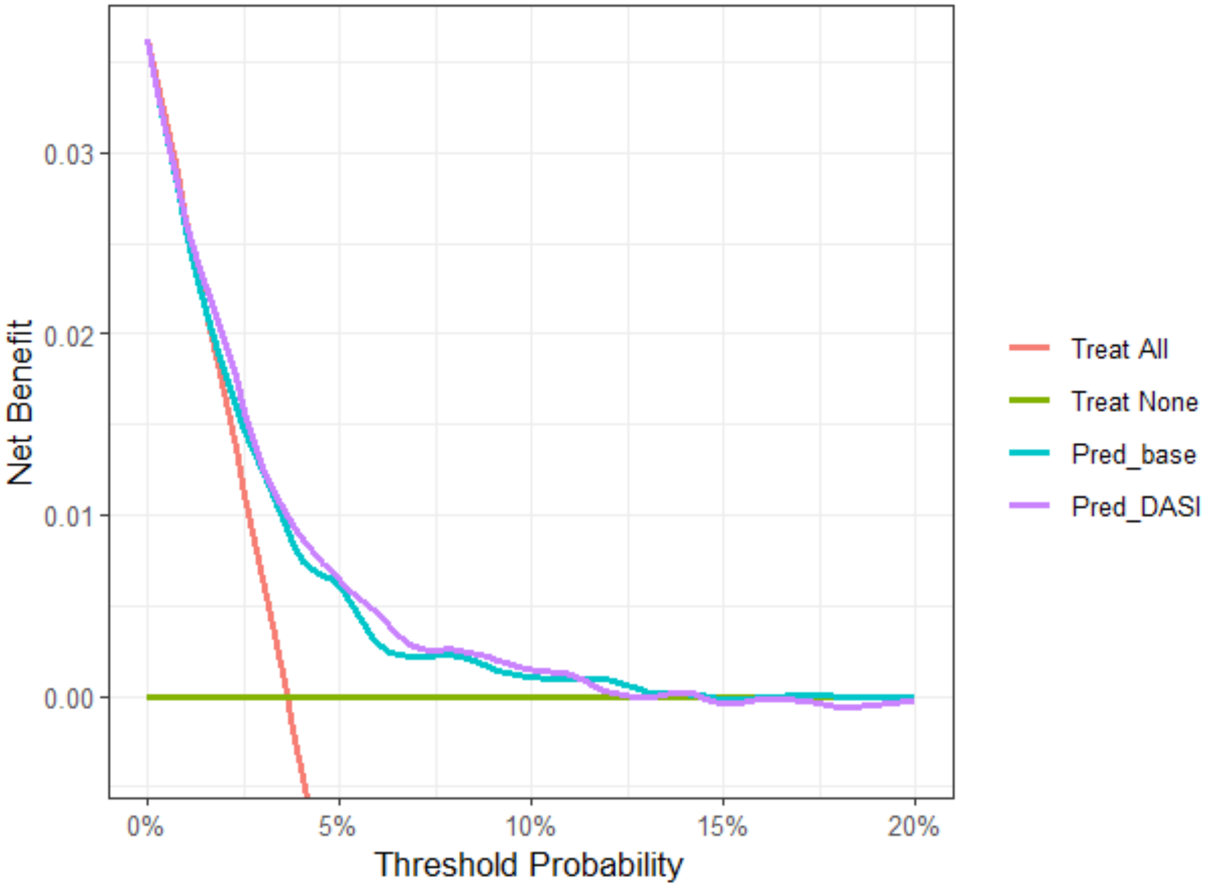

Legend: Decision curve analysis comparing clinical strategies for predicting 30-day postoperative major cardiac complications or death across a range of threshold probabilities (0% to 20%). The *Pred_base* model includes guideline-recommended clinical risk factors (age and RCRI), while *Pred_DASI* additionally incorporates the DASI. *Treat All* assumes all patients would receive a preventive intervention, and *Treat None* assumes no intervention. Both prediction models demonstrated modest net benefit over *Treat None* across threshold probabilities up to approximately 10%. However, the curves for *Pred_base* and *Pred_DASI* are nearly overlapping across all thresholds, indicating modest and inconsistent improvement from adding DASI. The *Treat All* strategy was inferior beyond thresholds of approximately 4%. At thresholds above 10%, none of the strategies yielded meaningful net benefit.

Figure S6: Net clinical benefit of adding DASI to age, Revised Cardiac Risk Index, and natriuretic peptide concentration (BNP or NT-proBNP) for predicting 30-day cardiac complications or death.

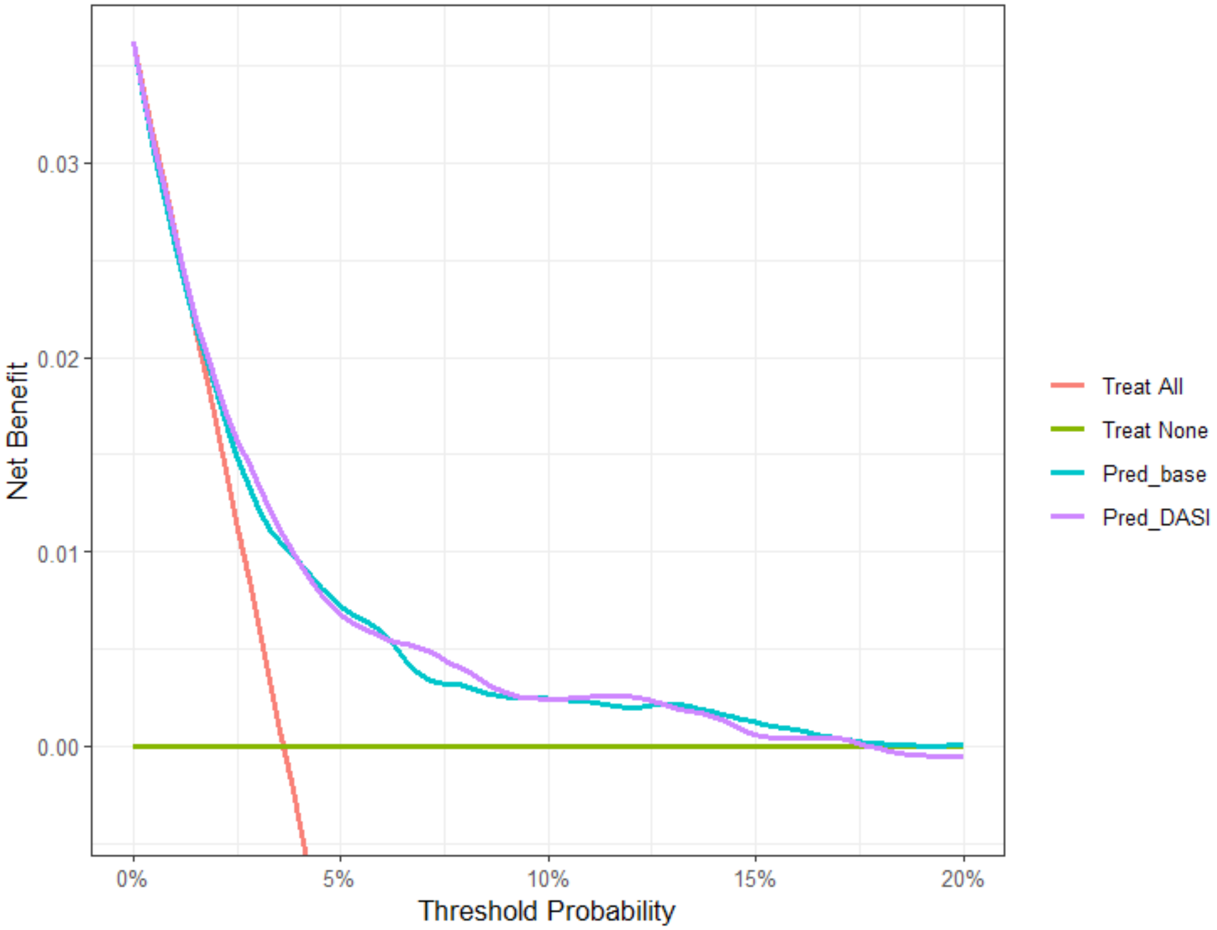

<u>Legend</u>: Decision curve analysis evaluating the net clinical benefit of adding DASI scores to prediction models that include age, RCRI, and preoperative natriuretic peptide concentrations (BNP or NT-proBNP) for estimating 30-day risk of major cardiac complications or death. *Pred_base* represents the model with age, RCRI, and natriuretic peptides, while *Pred_DASI* additionally includes DASI scores. *Treat All* assumes all patients receive a preventive intervention, whereas *Treat None* assumes no intervention. Both models demonstrated modest benefit over *Treat None* across threshold probabilities up to approximately 15%. However, the curves for *Pred_base* and *Pred_DASI* nearly overlap at all thresholds, indicating modest and inconsistent improvement when DASI is added to models already incorporating age, RCRI, and natriuretic peptides. The *Treat All* strategy was inferior above very low thresholds (4%), while no strategy showed appreciable benefit beyond threshold probabilities of 15%.

Figure S7: Association between continuous Duke Activity Status Index score and predicted log odds of the secondary outcome, modelled using restricted cubic splines

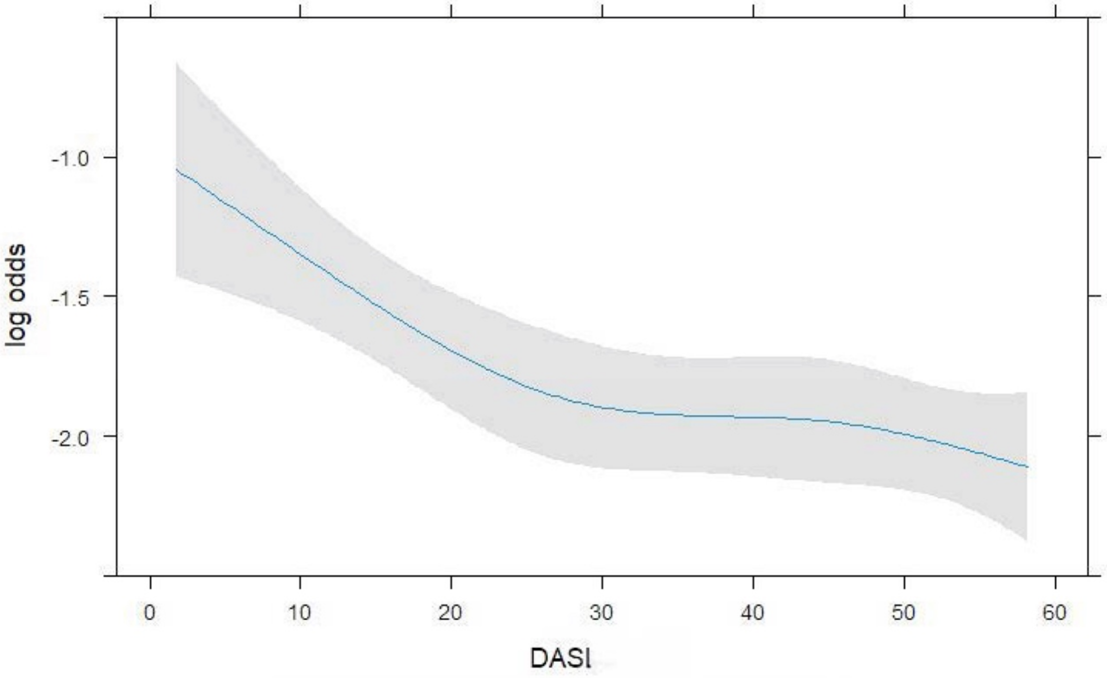

Legend: Predicted log odds of the secondary outcome (major postoperative complications) were derived from a logistic regression model including age, sex, surgery type, and DASI modelled using restricted cubic splines with four knots. Knots were placed at equally spaced quantiles between 0·05 and 0·95 (i.e., 5th, 35th, 65th, and 95th percentiles). The shaded area represents 95% confidence intervals. There was no evidence of significant nonlinearity for DASI (p=0·12 for nonlinear terms).

Figure S8: Net clinical benefit of adding DASI to age, sex, and surgery type for predicting major complications.

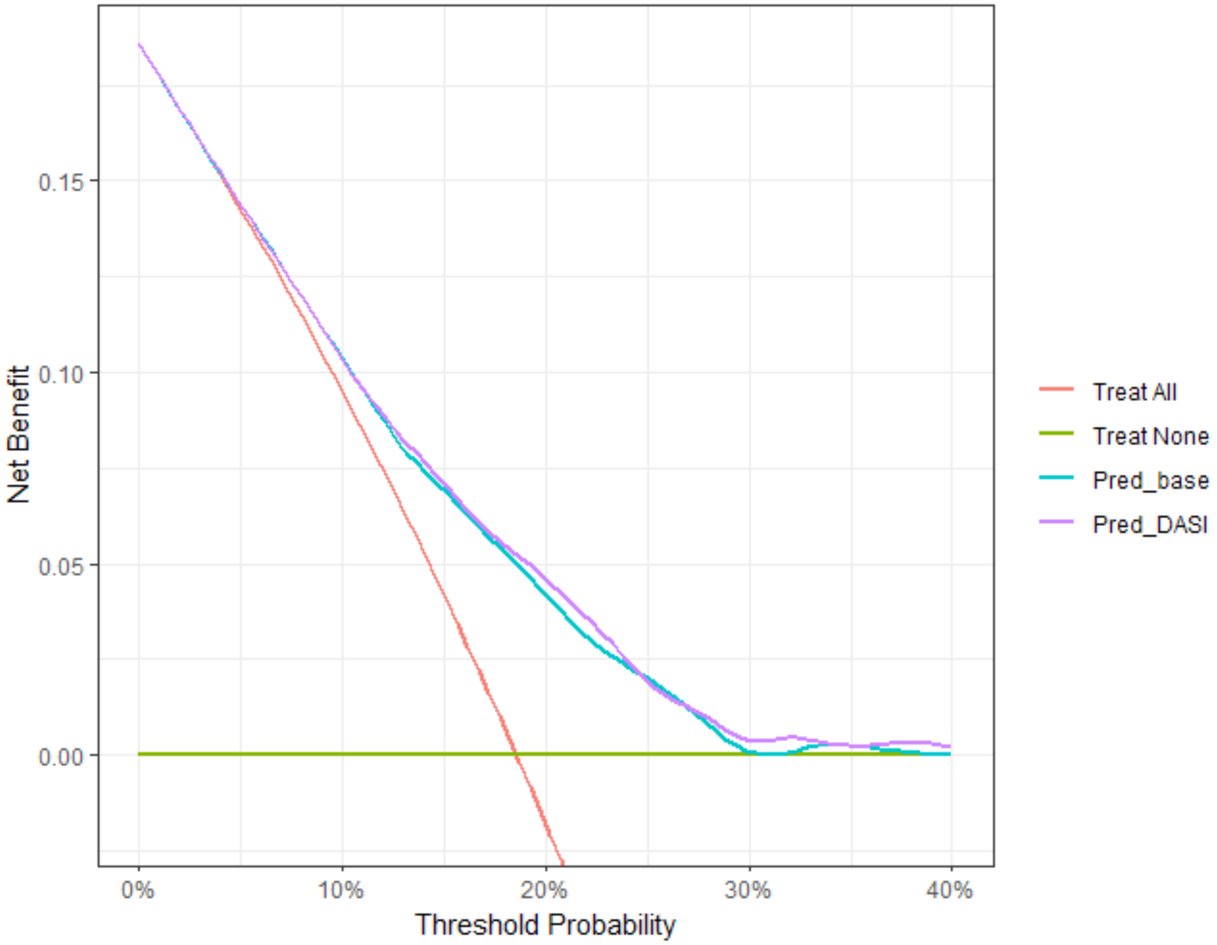

Legend: Decision curve analysis evaluating the net clinical benefit of adding DASI scores to prediction models that include age, sex, and surgery type for estimating the risk of major postoperative complications or death. *Pred_base* represents the model with age, sex, and surgery type only; *Pred_DASI* additionally includes DASI scores. *Treat All* assumes all patients receive intervention, while *Treat None* assumes no patient is treated. Both models demonstrated higher net benefit than a *Treat None* strategy across threshold probabilities up to approximately 25%. The *Treat All* strategy became inferior above low thresholds (5%). Incorporating DASI resulted in only marginal improvement, with the *Pred_DASI* and *Pred_base* curves largely overlapping across the entire range of thresholds. Net clinical utility diminished beyond thresholds of approximately 30%, at which point all strategies provided minimal benefit.

Table S1: Scoring scheme for the DASI

	Questionnaire Item	Points
1.	Can you take care of yourself, that is, eat dress, bathe or use the toilet?	2·75
2.	Can you walk indoors, such as around your house?	1·75
3.	Can you walk 200 yards on level ground?	2·75
4.	Can you climb a flight of stairs or walk up a hill?	5·50
5.	Can you run a short distance?	8·00
6.	Can you do light work around the house like dusting or washing dishes?	2·70
7.	Can you do moderate work around the house like vacuuming, sweeping floors, or carrying groceries?	3·50
8.	Can you do heavy work around the house like scrubbing floors or lifting or moving heavy furniture?	8·00
9.	Can you do yard work like raking leaves, weeding or pushing a power mower?	4·50
10.	Can you have sexual relations?	5·25
11.	Can you participate in moderate recreational activities like golf, bowling, dancing, doubles tennis, or throwing a ball?	6·00
12.	Can you participate in strenuous sports like swimming, singles tennis, football, basketball, or skiing?	7·50

Abbreviations: DASI, Duke Activity Status Index

Table S2: Definitions of postoperative events

Event	Definition
Unexpected admission to critical care unit	Unexpected admission to critical care unit, step-down unit, or high-dependency unit during index hospitalisation
Acute myocardial injury	Postoperative troponin concentration that (i) exceeds the 99th percentile upper reference limit for the assay, (ii) exceeds the preoperative troponin concentration, and (ii) is characterised by a rise and/or fall of troponin concentration values.[6]
Non-fatal cardiac arrest	Any successful resuscitation from ventricular fibrillation, sustained ventricular tachycardia, asystole, or pulseless electrical activity.
Myocardial infarction	Diagnosed by an Outcome Adjudication Committee using the Fourth Universal Definition of myocardial infarction.[6]
Acute heart failure	Presence of clinical (elevated jugular venous pressure, respiratory rales, crepitations or presence of third heart sound) and radiological (vascular redistribution or interstitial pulmonary oedema or frank pulmonary oedema) findings consistent with heart failure.[7]
Deep venous thrombosis [7]	Any of the following during index hospitalisation (1) persistent intraluminal filling defect on contrast venography; (2) ≥1 non-compressible venous segment on B-mode compression ultrasonography; and (3) clearly defined intraluminal filling defect on contrast enhanced computed tomography.
Pulmonary embolism [7]	Any of the following during index hospitalisation:<br>1. High probability ventilation/perfusion lung scan<br>2. Intraluminal filling defect of segmental or larger artery on a helical CT scan<br>3. Intraluminal filling defect on pulmonary angiography<br>4. A positive diagnostic test for DVT (e.g., positive compression ultrasound) PLUS one of the following:<br>a. Low or intermediate probability ventilation/perfusion lung scan<br>Non-diagnostic (sub-segmental defects or technically inadequate study) helical CT scan.
Acute stroke	Any new focal neurological deficit, suspected to be of vascular origin, with signs/symptoms lasting ≥24 hours.[7]
Transient ischemic attack	Any transient focal neurological deficit that lasted less than 24 hours and is thought to be vascular in origin.[7]
Respiratory failure	The need for tracheal reintubation and mechanical ventilation after extubation (within 30 days after surgery) or need for mechanical ventilation for >24 hours after surgery.[8]
Pneumonia [8,9]	≥2 serial chest radiographs with ≥1 of the following (one radiograph is sufficient for patients with no underlying pulmonary or cardiac disease): (1) new or progressive and persistent infiltrates; (2) consolidation; and (3) cavitation<br>AND<br>≥1 of following: (1) fever (>38 Celsius) with no other cause; (2) leukopenia (white cell count <4 x 10^9 per L) or leucocytosis (white cell count >12 x 109 per L); and (3) altered mental status in adults >70 y with no other cause<br>AND<br>≥2 of the following: (1) new onset of purulent sputum or change in character of sputum, or increased respiratory secretions, or increased suctioning requirements; (2) new onset or worsening cough, or dyspnoea, or tachypnoea, rales, or bronchial breath sounds; and (3) worsening gas exchange (hypoxemia, increased oxygen requirement, increased ventilator demand).
Superficial surgical site infection [10,11]	Infection within 30 days after the principal operative procedure<br>that involves only skin or subcutaneous tissue of the incision<br>AND ≥1 of the following is present: (1) purulent drainage from the superficial incision; (2) organisms isolated from an aseptically obtained culture of fluid or tissue from the superficial incision; and (3) superficial incision is deliberately opened by the surgeon<br>AND<br>≥1 of the following is present: (1) pain or tenderness; (2) localised swelling; (3) redness; and (4) heat.

Deep surgical site infection [10,11]	Infection within 30 days after the principal operative procedure<br>that involves deep soft tissues AND ≥1 of the following is present:<br>1. Purulent drainage from the deep incision but not from the organ/space component of the surgical site<br>2. A deep incision spontaneously dehisces or is deliberately opened by a surgeon when the patient has at least one of the following signs or symptoms: fever (>38° C), localised pain, or tenderness, unless the site is culture-negative<br>3. An abscess or other evidence of infection involving the deep incision is found on direct examination, during reoperation, or by histopathologic or radiologic examination<br>Diagnosis of a deep incision surgical site infection by a surgeon or attending physician.
Organ/space surgical site infection	Infection within 30 days after the principal operative procedure that involves any of the anatomy, other than the incision, which was opened or manipulated during the operation, AND ≥1 of the following is present:<br>1. Purulent drainage from a drain that is placed through a stab wound into the organ/space<br>2. Organisms isolated from an aseptically obtained culture of fluid or tissue in the organ/space<br>3. An abscess or other evidence of infection involving the organ/space that is found on direct examination, during reoperation, or by histopathologic or radiologic examination<br>Diagnosis of an organ/space surgical site infection by a surgeon or attending physician.
Wound disruption	Spontaneous reopening of a surgically closed wound that occurs within 30 days after the principal operative procedure AND one of the following criteria below is present:<br>1. Abdominal site: loss of the integrity of fascial closure (or whatever other closure was performed instead)<br>2. Other surgical sites: total breakdown of the surgical closure compromising the integrity of the procedure.
New requirement for dialysis	New requirement for dialysis during index hospitalisation.
Reoperation	Return to operating room within index hospitalisation.
Complication severity [4]	Most severe postoperative complication is further classified as:<br>• mild (resulted in only temporary harm and did not require clinical treatment)<br>• moderate (required clinical treatment but without causing significant prolongation of hospital stay or permanent functional limitation)<br>• severe (required clinical treatment and resulted in significant prolongation of hospital stay and/or permanent functional limitation)<br>• fatal (resulted in death).

Table S3: Scoring scheme for the four-question (modified) DASI (M-DASI-4Q)

Questionnaire Item [12,13]		Points
1.	Can you climb a flight of stairs or walk up a hill?	1
2.	Can you do heavy work around the house like scrubbing floors or lifting or moving heavy furniture?	1
3.	Can you do yard work like raking leaves, weeding or pushing a power mower?	1
4.	Can you participate in strenuous sports like swimming, singles tennis, football, basketball, or skiing?	1

Abbreviations: M-DASI-4Q, four-question (modified) Duke Activity Status Index

Table S4: Variables included in the multiple imputation model

Variable	Missing Values* N(%)
Study (METS vs. FIT After Surgery)	0
Age	0
Female sex	0
Height	146 (4·2%)
Weight	145 (4·2%)
Ischaemic heart disease	0
Heart failure	0
Atrial fibrillation	0
Cerebrovascular disease	0
Peripheral artery disease	0
Diabetes mellitus	0
Hypertension	0
Renal replacement therapy	0
Cancer	0
ASA-PS class	14 (0·4%)
DASI score	20 (0·6%)
M_DASI_4Q	20 (0·6%)
Preoperative creatinine concentration	178 (5·1%)
Preoperative haemoglobin concentration	214 (6·1%)
Elevated preoperative natriuretic peptide	322 (9·2%)
Elevated preoperative troponin	509 (14·6%)
Surgery type	0
Peak postoperative creatinine concentration on days 1 to 3	341 (9·8%)
Postoperative in-hospital length of stay	2 (0·06%)
Postoperative complication severity	2 (0·06%)
Postoperative myocardial infarction	1 (0·03%)
Postoperative non-fatal cardiac arrest	1 (0·03%)
Postoperative death within 30 days after surgery	16 (0·5%)

Abbreviations: ASA-PS, American Society of Anesthesiologists Physical Status; DASI, Duke Activity Status Index; M-DASI-4Q, four-question (modified) Duke Activity Status Index

* Missing values were handled using multiple imputation by chained equations (m=30), with each incomplete variable imputed iteratively using predictive mean matching. The imputation model included all variables listed in table S4, including study and outcome variables. Outcome variables were included as predictors in the imputation model, but individuals with missing outcomes were excluded from the analytic models.

Table S5: Component overlap within the 30-day primary composite outcome

Individual Clinical Events	n	Proportion of 126 Primary Outcome Events (%)
Myocardial infarction only	98	77·8
Death only	16	12·7
Nonfatal cardiac arrest only	6	4·5
Myocardial infarction + death	2	1·6
Myocardial infarction + nonfatal cardiac arrest	2	1·6
Nonfatal cardiac arrest + death	1	0·8
Myocardial infarction + nonfatal cardiac arrest + death	1	0·8

Table S6: Bivariate comparisons between patients who did versus did not experience the secondary outcome (in-hospital major postoperative complication)

	Major Complications (n=647)	No Major Complications (n=2836)	Missing Outcome (n=2)
**Demographics**			
Median age (IQR) – y	71 (66–76)	70 (65–74)	66·0 (57–75)
Female sex – no. (%)	252 (39%)	1198 (42%)	1 (50%)
**Baseline Assessments**			
Median DASI (IQR)	34·7 (19·0–50·2)	37·0 (23·5–50·7)	39·0 (25·0–53·0)
Missing DASI	2	18	0
M-DASI-4Q			
0 points	74 (11%)	265 (9·4%)	0 (0%)
1 point	175 (27%)	683 (24%)	1 (50%)
2 points	111 (17%)	512 (18%)	0 (0%)
3 points	66 (26%)	745 (26%)	0 (0%)
4 points	119 (18%)	613 (22%)	1 (50%)
Missing	2	18	0
Smoking status – no. (%)			
Any smoking in prior year	103 (16%)	403 (14%)	1 (50%)
Remote smoking history	288 (45%)	1107 (39%)	1 (50%)
Non-smoker	256 (40%)	1326 (47%)	0 (0%)
**Comorbid Disease – no. (%)**			
Ischemic heart disease	120 (19%)	425 (15%)	0 (0%)
Heart failure	28 (4·3%)	68 (2·4%)	0 (0%)
Atrial fibrillation	74 (11%)	193 (6·8%)	0 (0%)
Cerebrovascular disease	41 (6·3%)	151 (5·3%)	1 (50%)
Peripheral artery disease	39 (6·0%)	144 (5·1%)	0 (0%)
Diabetes mellitus requiring treatment	144 (22%)	503 (18%)	1 (50%)
Hypertension	381 (59%)	1640 (58%)	2 (100%)
Renal replacement therapy	11 (1·7%)	28 (1·0%)	0 (0%)
Preoperative renal insufficiency *	28 (4·5%)	50 (1·9%)	0 (0%)
Missing †			
Obstructive pulmonary disease	106 (16%)	408 (14%)	1 (50%)
Cancer			
Unrelated to planned surgery	30 (4·6%)	190 (6·7%)	0 (0%)
Planned cancer surgery	372 (57%)	1196 (42%)	2 (100%)
Arthritis	187 (29%)	1136 (40%)	1 (50%)
ASA-PS class			
I	13 (2·0%)	100 (3·5%)	0 (0%)
II	194 (30%)	964 (34%)	0 (0%)
III	311 (49%)	1442 (51%)	2 (100%)
IV	120 (19%)	325 (11%)	0 (0%)
Missing	9	5	0
Revised Cardiac Risk Index			
0 points	114 (18%)	1092 (41%)	0 (0%)
1 point	335 (53%)	1109 (41%)	0 (0%)
2 points	120 (19%)	370 (14%)	1 (100%)
≥3 points	58 (9·3%)	108 (4·0%)	0 (0%)
Missing †	20	157	1
Elevated natriuretic peptide ‡	204 (35%)	607 (24%)	0 (0%)
Missing	59	262	1
**Operative Characteristics**			
Surgery type – no. (%)			
Vascular	27 (4·2%)	113 (4·0%)	0 (0%)
Intra-thoracic	30 (4·6%)	150 (5·3%)	1 (50%)
Intra-peritoneal	382 (59%)	936 (33%)	1 (50%)
Major spine	56 (8·7%)	334 (12%)	0 (0%)
Orthopaedic	14 (2·2%)	357 (13%)	0 (0%)
Urologic or gynaecologic	109 (17%)	744 (26%)	0 (0%)
Head-and-neck	24 (3·7%)	152 (5·4%)	0 (0%)
Other	5 (0·8%)	50 (1·8%)	0 (0%)

Abbreviations: ASA-PS, American Society of Anesthesiologists Physical Status; DASI, Duke Activity Status Index; M-DASI-4Q, four-question (modified) Duke Activity Status Index

* Defined as preoperative creatinine concentration ≥177 μmol/L or dialysis-dependence
† Related to missing preoperative creatinine concentration data in 178 patients
‡ Defined as BNP ≥92 ng/L or NT-proBNP ≥200 ng/L

Table S7: Adjusted association of DASI with primary and secondary outcomes

Model	Risk Factor	aOR (95% CI)	p-value
**Outcome: 30-Day Major Cardiac Complication or Death**			
**Model 1**			
Age, RCRI, DASI	Age (per 5-y increase)	1·23 (1·08–1·40)	0·001
	RCRI		0·004
	0 points	Reference	
	1 point	1·10 (0·70–1·73)	
	2 points	1·62 (0·95–2·74)	
	≥3 points	3·03(1·64–5·59)	
	DASI (per 5-unit decrease)	1·11 (1·04–1·18)	0·001
**Model 2**			
Age, RCRI, biomarker, DASI	Age (per 5-y increase)	1·15 (1·01–1·31)	0·04
	RCRI		0·03
	0 points	Reference	
	1 point	1·02 (0·65–1·61)	
	2 points	1·42 (0·84–2·42)	
	≥3 points	2·48 (1·33–4·61)	
	Elevated BNP or NT-proBNP*	2·11 (1·40–3·17)	<0·001
	DASI (per 5-unit decrease)	1·09 (1·02–1·16)	0·009
**Sensitivity Analysis**			
Age, RCRI, log(NP), DASI	Age (per 5-y increase)	1·11 (0·97–1·27)	0·12
	RCRI		0·12
	0 points	Reference	
	1 point	0·99 (0·63–1·56)	
	2 points	1·32 (0·77–2·25)	
	≥3 points	2·02 (1·06–3·84)	
	Log (harmonised NT-proBNP)*‡	1·45 (1·23–1·71)	<0·001
	DASI (per 5-unit decrease)	1·09 (1·02–1·16)	0·01
**Outcome: Major Complication**			
**Model 3**			
Age, sex, surgery, DASI	Age (per 5-y increase)	1·08 (1·03–1·14)	0·004
	Biological sex		
	Male	Reference	
	Female	0·81 (0·67–0·97)	0·03
	Surgery type		<0·001
	Other procedure	Reference	
	Lower limb joint replacement	0·25 (0·14–0·44)	
	Elevated risk procedure §	2·65 (2·20–3·20)	
	DASI (per 5-unit decrease)	1·07 (1·04–1·10)	<0·001

Abbreviations: aOR, adjusted odds ratio; DASI, Duke Activity Status Index; NP, natriuretic peptide; RCRI, Revised Cardiac Risk Index

* Defined as BNP ≥92 ng/L or NT-proBNP ≥200 ng/L.[14]

† Natriuretic peptide concentration was modelled as a continuous variable using a harmonised, log-transformed NT-proBNP scale. For participants with BNP measurements, values were converted to NT-proBNP using a validated conversion equation.[15]

‡ The aOR corresponds to a 1-unit increase in log(NT-proBNP), equivalent to an approximately 2.7-fold increase in NT-proBNP concentration.

§ Defined as intraperitoneal, intrathoracic, or supra-inguinal vascular procedures.[16]

Table S8: Additional predictive performance of M-DASI-4Q with respect to primary and secondary outcomes

Covariates in Model	c-index	Likelihood Ratio Test	Fraction of New Predictive Information	Net Reclassification Improvement Index		
				Events	Non-Events	Overall (95% CI)
**Primary Outcome: 30-Day Major Cardiac Complication or Death**						
**Model 1**						
Age, RCRI	0·68 (0·63–0·73)					
+ M-DASI-4Q	0·69 (0·65–0·74)	p=0·04	15·6%	0·16 (-0·06–0·38)	0·11 (0–0·22)	0·26 (0·08–0·44)
**Model 2**						
Age, RCRI, abnormal NP*	0·70 (0·65–0·74)					
+ M-DASI-4Q	N.A.	p=0·13	N.A.	N.A.	N.A.	N.A.
**Secondary Outcome: All-Cause Major Postoperative Complication**						
**Model 3**						
Age, sex, surgery type	0·67 (0·65–0·69)					
+ M-DASI-4Q	0·68 (0·66–0·70)	p=0·001	6·6%	-0·14 (-0·22– -0·07)	0·28 (0·24–0·32)	0·14 (0·05–0·22)

Abbreviations: M-DASI-4Q, four-question (modified) Duke Activity Status Index; NP, natriuretic peptide

* Defined as BNP ≥92 ng/L or NT-proBNP ≥200 ng/L

Table S9: Adjusted association of M-DASI-4Q with primary and secondary outcomes

Model	Risk Factor	aOR (95% CI)	p-value
**Outcome: 30-Day Major Cardiac Complication or Death**			
**Model 1**			
Age, RCRI, M-DASI-4Q	Age (per 5-y increase)	1·25 (1·10–1·42)	0·001
	RCRI		0·004
	0 points	Reference	
	1 point	1·08 (0·36–1·69)	
	2 points	1·61 (0·95–2·72)	
	≥3 points	2·97 (1·61–5·46)	
	M-DASI-4Q		0·04
	4 points	Reference	
	3 points	0·72 (0·38–1·38)	
	2 points	1·16 (0·62–2·19)	
	1 point	1·46 (0·82–2·61)	
	0 points	1·77 (0·91–3·45)	
**Outcome: All-Cause Major Complication**			
**Model 3**			
Age, sex, surgery, M-DASI-4Q	Age (per 5-y increase)	1·10 (1·04–1·15)	<0·001
	Biological sex		
	Male	Reference	
	Female	0·82 (0·68–0·99)	0·04
	Surgery type		<0·001
	Other procedure	Reference	
	Lower limb joint replacement	0·25 (0·15–0·44)	
	Elevated risk procedure†	2·63 (2·18–3·18)	
	M-DASI-4Q		0·001
	4 points	Reference	
	3 points	1·11 (0·85–1·45)	
	2 points	1·19 (0·88–1·60)	
	1 point	1·50 (1·14–1·97)	
	0 points	1·89 (1·34–2·67)	

Abbreviations: aOR, adjusted odds ratio; M-DASI-4Q, four-question (modified) Duke Activity Status Index RCRI, Revised Cardiac Risk Index

* Defined as BNP ≥92 ng/L or NT-proBNP ≥200 ng/L.[14]
† Defined as intraperitoneal, intrathoracic, or supra-inguinal vascular procedures.[16]

Appendix References

1 Wijeysundera DN, Pearse RM, Shulman MA, *et al.* Measurement of Exercise Tolerance before Surgery (METS) study: a protocol for an international multicentre prospective cohort study of cardiopulmonary exercise testing prior to major non-cardiac surgery. *BMJ Open* 2016; **6**: e010359.
2 Hlatky MA, Boineau RE, Higginbotham MB, *et al.* A brief self-administered questionnaire to determine functional capacity (the Duke Activity Status Index). *Am J Cardiol* 1989; **64**: 651–4.
3 Thygesen K, Alpert JS, Jaffe AS, *et al.* Third universal definition of myocardial infarction. *Circulation* 2012; **126**: 2020–35.
4 International Surgical Outcomes Study (ISOS) group. Prospective observational cohort study on grading the severity of postoperative complications in global surgery research. *Br J Surg* 2019; **106**: e73–80.
5 Wijeysundera DN, Alibhai SMH, Ladha KS, *et al.* Functional Improvement Trajectories After Surgery (FIT After Surgery) study: protocol for a multicentre prospective cohort study to evaluate significant new disability after major surgery in older adults. *BMJ Open* 2022; **12**: e062524.
6 Thygesen K, Alpert JS, Jaffe AS, *et al.* Fourth universal definition of myocardial infarction (2018). *Eur Heart J* 2019; **40**: 237–69.
7 Botto F, Alonso-Coello P, Chan MT, *et al.* Myocardial injury after noncardiac surgery: a large, international, prospective cohort study establishing diagnostic criteria, characteristics, predictors, and 30-day outcomes. *Anesthesiology* 2014; **120**: 564–78.
8 Abbott TEF, Fowler AJ, Pelosi P, *et al.* A systematic review and consensus definitions for standardised end-points in perioperative medicine: pulmonary complications. *Br J Anaesth* 2018; **120**: 1066–79.
9 Horan TC, Andrus M, Dudeck MA. CDC/NHSN surveillance definition of health care-associated infection and criteria for specific types of infections in the acute care setting. *Am J Infect Control* 2008; **36**: 309–32.
10 Horan TC, Gaynes RP, Martone WJ, Jarvis WR, Emori TG. CDC definitions of nosocomial surgical site infections, 1992: a modification of CDC definitions of surgical wound infections. *Infect Control Hosp Epidemiol* 1992; **13**: 606–8.
11 Barnes J, Hunter J, Harris S, *et al.* Systematic review and consensus definitions for the Standardised Endpoints in Perioperative Medicine (StEP) initiative: infection and sepsis. *Br J Anaesth* 2019; **122**: 500–8.
12 Riedel B, Li MH, Lee CHA, *et al.* A simplified (modified) Duke Activity Status Index (M-DASI) to characterise functional capacity: a secondary analysis of the Measurement of Exercise Tolerance before Surgery (METS) study. *Br J Anaesth* 2021; **126**: 181–90.
13 Lee CHA, Ismail H, Ho KM, Riedel B, Schier R. Thresholds of functional capacity using the four-question (modified) Duke Activity Status Index (M-DASI-4Q) as a screening tool: observations from the Measurement of Exercise Tolerance Before Surgery (METS) study. *Br J Anaesth* 2021; **126**: e92–4.
14 Duceppe E, Mills NL, Mueller C, Study Group on Biomarkers of the ESC Association for Acute Cardiovascular Care. How to use natriuretic peptides in non-cardiac surgery. *Eur Heart J Acute Cardiovasc Care* 2024; **13**: 380–2.
15 Kasahara S, Sakata Y, Nochioka K, *et al.* Conversion formula from B-type natriuretic peptide to N-terminal proBNP values in patients with cardiovascular diseases. *Int J Cardiol* 2019; **280**: 184–9.
16 Lee TH, Marcantonio ER, Mangione CM, *et al.* Derivation and prospective validation of a simple index for prediction of cardiac risk of major noncardiac surgery. *Circulation* 1999; **100**: 1043–9.
